# Compound Discovery and Structure-Activity Relationship Study of Neoantimycins Against Drug-Resistant Cancer Cells

**DOI:** 10.3389/fchem.2019.00481

**Published:** 2019-07-05

**Authors:** Xiao Lin, Yongjun Zhou, Liyun Liu, Hongrui Zhu, Yeping Chen, Shuping Wang, Fan Sun, Ling Chai, Buming Liu, Shihai Xu, Hou-Wen Lin

**Affiliations:** ^1^College of Chemistry and Materials, Jinan University, Guangzhou, China; ^2^College of Pharmacy, Jinan University, Guangzhou, China; ^3^State Key Laboratory of Oncogenes and Related Genes, Department of Pharmacy, Research Center for Marine Drugs, School of Medicine, Ren Ji Hospital, Shanghai Jiao Tong University, Shanghai, China; ^4^Guangxi Key Laboratory of Traditional Chinese Medicine Quality Standards, Guangxi Institute of Traditional Medical and Pharmaceutical Sciences, Nanning, China

**Keywords:** neoantimycins, streptomyces conglobatus, structural characterization, enzymatically conversion, absolute structure, drug-resistant cancer cell lines, structure-activity relationship, apoptosis

## Abstract

Four neoantimycins H-K (1–4) with C1-keto, including the new ones (1–2), were isolated from the culture of *Streptomyces conglobatus* RJ8. After enzymatically converting into their respective reduced type derivatives (5–8) *in vitro*, the absolute structures of 1–8 were established/reconfirmed by analyzing hydrolyzed components. The obtained NATs (4, 7, and 8) exhibited excellent cytotoxicity against drug-resistant colon and gastric cancer cells but low toxicity in the noncancerous cell. Further SAR investigation suggested that C1-hydroxyl, C9-isobutyl, and *N*-formyl contribute to the antiproliferation remarkably.

## Introduction

Neoantimycins (NATs), a ring-expanded subfamily of antimycin, were thrust into the limelight in recent years (Lim et al., 2016; Liu et al., 2016; Awakawa et al., 2018; Skyrud et al., 2018). Unlike antimycins, inducing cancer cell apoptosis by inhibiting Bcl2/Bcl-xL-related anti-apoptotic proteins or mitochondrial electron transport, NATs were characterized as down-regulators of GRP-78 (Umeda et al., 2005; Izumikawa et al., 2007; Ueda et al., 2008; Kozone et al., 2009). GRP-78 was reported to promote protein folding in the endoplasmic reticulum and provides resistance to chemotherapy in cancer treatment, hence, inhibiting GRP-78 expression may be useful for the anti-cancer treatment of drug resistance (Lee, 2014). NATs were also documented as potent inhibitors of GTPase K-Ras plasma membrane (PM) localization with nanomolar potency (1–10 nM) (Salim et al., 2014). The inhibition of Ras PM localization could block all oncogenic activity (Baines et al., 2011), which implies NATs are promising lead compounds for treating drug-resistant cancer related to P-glycoprotein (P-gp) mediated drug efflux, and it was found that NATs exhibited significant cytotoxicity toward SW620 Ad300 cell line in which P-gp was overexpressed (Salim et al., 2014). However, the number of NATs discovered to date is limited, and among the 13 reported NATs, a handful of them didn't exhibit excellent anticancer effects, For example, SW-163 A and B showed no cytotoxicity against K562 and KB cells (Takahashi et al., 2001); NAT-D (5), E (6), and F (8) hardly had any significant antiproliferative activity against the HT-29 cell line (Li et al., 2013). The small drug reservoir hampers a detailed investigation of NATs, therefore, it is meaningful to fashion more NATs by biosynthetic engineering (Awakawa et al., 2018) or chemical synthesis (Manaviazar et al., 2016) and investigate the structure-activity relationship (SAR) of NATs against cancer cells.

Meanwhile, the stereochemistry is also regarded as an important factor for SAR of NATs. JBIR-04 and−05 isolated from Streptomyces violaceoniger 4541-SVS are two NATs similar to prunustatin A and 5, respectively, in the planar structure. JBIR-04 and−05 had negative optical value (−28.6° and −17.3°, respectively) and inhibited the expression of GRP78, while 5 had positive value (+27°) and did not show any cytotoxic activity (Izumikawa et al., 2007; Li et al., 2013). In addition, Manaviazar et al. reported the synthesis of JBIR-04 diastereoisomer ([α]_D_ +19.4°) and suspected that the different stereostructure of NATs would affect the down regulation of GRP78 (Manaviazar et al., 2016). The above evidence strongly implies that the stereochemical determination is pivotal to the investigation of NATs. However, the stereochemistry assignment for most NATs was undefined or incomplete. To the best of our knowledge, only six NATs' absolute configurations (3, 7, 8, NAT-G, prunustatin A, and SW163A) were established by analyzing chemically degraded components (Umeda et al., 2007; Salim et al., 2014). Even so, for NATs with keto at C-1 (e.g., 3 and prunustatin A), their absolute configural assignments were more troublesome since the racemization could occur at the C-2 during hydrolysis (Umeda et al., 2007), and their absolute configurations were deduced indirectly: Compound 7 and SW-163A were subjected to Dess-Martin oxidation to yield oxidized products at C-1, whose identical spectroscopic data with 3 (Salim et al., 2014) and prunustatin A (Umeda et al., 2007) confirmed the absolute configuration of NATs with C1-keto. Nevertheless, unexpected byproducts and isomers would occur during this chemical derivatization (Salim et al., 2014), which adds to the difficulty of the configurational assignment.

In our course of biosynthesis programming for engineering and producing more NATs, we isolated two new NATs (1 and 2) with C1-keto, along with two known ones (3 and 4) from a mutant *Streptomyces conglobatu*, in which C-1 keto derivatives were accumulated (Zhou et al., 2018). Since it is necessary to determine the stereochemistry in every NATs, a developed enzymatic conversion was applied *in vitro* to generate the C1-hydroxyl derivatives, NAT-D (5), E (6), A (7), and F (8), for confirming the absolute structure of 1–4. With eight NATs, a substantial SAR study of NATs toward the drug-resistant cancer cell lines is also presented. What follows is an account of our chemical and biological studies into NATs.

## Materials and Methods

### General Experimental Procedures

Optical rotations were measured with an Autopol VI, Serial #91003, and UV spectra on a Persee Tu-1,950 spectrophotometer. NMR spectra were obtained from an Agilent DD2 600 MHz NMR spectrometer and a Brucker AVANCE III HD 600 MHz NMR spectrometer. The ESI-MS analysis was performed on a Waters purification system coupled with a Waters Acquity QDa detector. The High-resolution TOF-ESI-MS spectra were acquired with a Waters XeVO G2-XS Q-TOF mass spectrometer. Preparative HPLC and semi-preparative HPLC were performed, respectively, on YMC-Pack Pro C_18_ RS (20 × 250 mm, 5 μm) column and Waters Xbridge Prep C_18_ (10 × 250 mm, 5 μm) column, coupled with a Waters 1,525 separation module and a Waters 2,998 photodiode array detector.

### Mediums and Culture Conditions

*S*. *conglobatus* RJ8 was grown in TSBY medium (3% tryptone soy broth, 10.3% sucrose, 0.5% yeast extract) at 30°C and 200 rpm to produce spore suspension. The suspension (5%) was added to the seed medium and then the production medium (SGC medium), which contain 3% soybean flour (the supernatant after first autoclaving was used), 5% glucose (autoclaved separately), 0.5% CaCO_3_, 5 mg/L CoCl_2_·6H_2_O (omitted in the production medium), and 0.2% (v/v) anti-foam. Fermentation was carried out by inoculating 150 mL medium in a 500 mL conical flask fitted with a metal spring at 30°C, 220 rpm for 3 and 5 days for seed and production culture, respectively.

### Extraction and Isolation

The culture broth (12 L) of *S*. *conglobatus* mutant RJ8 was extracted three times with an equal volume (12 L) of EtOAc at room temperature after adjusting pH to 6 with formic acid. The organic extract was concentrated under vacuum to afford an EtOAc extract (8.1 g), which was then dissolved in methanol (300 mL) and extracted with hexane (300 mL) 3 times for degreasing. The entire MeOH-soluble extract (6.6 g) was subjected to vacuum liquid chromatography (VLC) on silica gel (200–300 mesh) using a stepwise gradient elution of dichloromethane-MeOH (from 50:1 to 0:1, v/v) to yield six fractions (B1–B6). Fraction B1 (2.5 g) was subsequently passed through an ODS chromatography column eluted with a gradient of aqueous acetonitrile (from 30 to 100%) to give 14 fractions (B1A–B1N). Fraction B1J (1.2 g) was further fractionated over a silica flash column eluted with petroleum ether (PE)-EtOAc-MeOH (from 8:1:0 to 0:0:1, v/v/v) to give 10 subfractions. Subfraction B1J5 was purified by semi-preparative HPLC (Waters Xbridge C18, 10 × 250 mm, 3 mL/min) under isocratic conditions using 70% aqueous acetonitrile with 0.1% formic acid as buffer to yield neoantimycin J (1) (8.9 mg, t_R_ = 29.0 min) and K (2) (7.4 mg, t_R_ = 23.0 min). Subfraction B1J7 (1.0 g) was purified by preparative HPLC (YMC-Park C18, 20 × 250 mm, 5 μm, 8 mL/min) using 85% aqueous acetonitrile with 0.1% formic acid to afford neoantimycin H (3) (400 mg, t_R_ = 18.5 min) and three other subfractions (B1J7P1-B1J7P3). Subfraction B1J7P3 was further purified by semi-preparative HPLC (Waters Xbridge C18, 10 × 250 mm, 3 mL/min) under isocratic conditions using 70% aqueous acetonitrile with 0.1% formic acid as the buffer to yield neoantimycin I (4) (62 mg, t_R_ = 19.0 min).

### The Expression and Purification of NatE Protein

NatE protein (13 mg) was expressed and purified following the protocol published in our previous study (Zhou et al., 2018). The 1144 bp PCR product of the *natE* gene was amplified from genomic DNA with primers nat7P-S and nat7P-A (Table 1). The C-terminal 6 × His tag was introduced by the primer nat7P-A. The PCR product was digested with NdeI and XhoI and then introduced to the same sites of pET29a. The resulting plasmid pRJ35 was introduced into *E. coli* BL21 (DE3) plysS for protein expression and purification. A single colony of *E. coli* BL21 (DE3) plysS was inoculated into 5 mL of LB medium with 50 μg/mL kanamycin and grown at 37°C for 12 h. 1 mL of the culture was inoculated into 1 L LB medium with 50 μg/mL kanamycin and incubated at 37°C, 200 rpm until the A_600_ value reached to 0.6~0.8. After that, isopropyl-β-D-thiogalactopyranoside was added to 0.2 mM final concentration and giving a further incubation at 22°C for 15 h to induce protein expression. Cells were pelleted at 11,325 × *g* for 5 min, resuspended in lysis buffer (50 mM Tris HCl, 0.3 M NaCl, pH 7.2) and lysed by sonication. The total lysate was centrifuged at 34,925 × *g* for 25 min, and the supernatant was filtered through a 0.45 μm membrane before loading onto a His-Bind affinity column (1 mL bed volume). The column was then washed with 10 column volumes of lysis buffer. Bound proteins were eluted by stepwise increases in the concentration of imidazole (up to 500 mM). The proteins were concentrated and the buffer was exchanged into 50 mM Tris.HCl (pH8.0) using Amicon Ultra-4 concentrators (Millipore) fitted with a filter of 10 kDa cut off. The yield of NatE was 8 mg/L. The purified proteins were analyzed by 4–12% Bis-Tris Gel SDS-PAGE. Protein concentrations were measured using a NanoDrop 1,000 spectrophotometer.

**Table 1:** Primers used in this study.

**Clone of** ***natE*** **gene based on pET29a for protein expression with a C-terminal 6** **×** **His tag**		
nat7P-S	ATATACATATGAGGCTGCTGATCCTCGGCGGCA	1,144 bp product, NdeI and XhoI site are underlined
nat7P-A	ATTATCTCGAGTCAATGATGATGATGATGATGTGACACTCGGCTTC	

### The Conversion and Purification of Compounds 5–8

The *in vitro* enzymatic conversion for 7 was carried out by combining 4.2 mg NatE, 15 mg neoantimycin H (3), 1.4 mL NADPH (1 mM), and 3.5 mL DMSO in 35 mL of 50 mM Tris HCl (pH8.0) buffer. After 30 min incubation at 30°C, the reaction was quenched and extracted by an equal volume of EtOAc three times. The organic extract was combined and concentrated under vacuum to afford an EtOAc extract, which was subsequently redissolved in MeOH and purified using semi-preparative HPLC (Waters Xbridge C18, 10 × 250 mm, 3 mL/min) using 70% aqueous acetonitrile with 0.1% formic acid to afford 7 (10 mg, t_R_ = 16.0 min). Analogously, 5, 6, and 8 were converted from 1, 2, and 4 by NatE in smaller scale, semi-preparative HPLC (Waters Xbridge C18, 10 × 250 mm, 3 mL/min) was adapted to afford 5 (1.1 mg), 6 (2.0 mg), and 8 (4.0 mg), respectively.

### Characterization of Compounds 1–8

**Neoantimycin J (1)**. Pale yellow amorphous solid; ${[\alpha ]}_{\text{D}}^{25}$ + 24.0 (*c* = 0.5, CHCl_3_); UV (MeOH) λmax (log ε) 207 (4.34), 227 (4.30), 331 (3.56) nm; NMR data see Table S2, and Figure S1; HRESIMS *m/z* 669.3054 [M+H]^+^ (calcd for C_35_H_45_N_2_O_11_, 669.3023) (Table S1).
**Neoantimycin K (2)**. Pale yellow amorphous solid; ${[\alpha ]}_{\text{D}}^{25}$ + 31.3 (*c* = 0.5, CHCl_3_); UV (MeOH) λmax (log ε) 207 (4.06), 227 (3.96), 331 (3.34) nm; NMR data see Table S3, and Figure S2; HRESIMS *m/z* 655.2896 [M+H]^+^ (calcd for C_34_H_43_N_2_O_11_, 655.2867) (Table S1).
**Neoantimycin H (3)**. Pale yellow amorphous solid; ${[\alpha ]}_{\text{D}}^{25}$ + 57.5 (*c* = 0.25, CHCl_3_); UV (MeOH) λmax (log ε) 223 (4.25), 321 (3.45) nm; NMR data see Table S4, and Figure S3; HRESIMS *m/z* 697.2987 [M+H]^+^ (calcd for C_36_H_45_N_2_O_12_, 697.2972) (Table S1).
**Neoantimycin I (4)**. Pale yellow amorphous solid; ${[\alpha ]}_{\text{D}}^{25}$ + 68.3 (*c* = 0.25, CHCl_3_); UV (MeOH) λmax (log ε) 223 (4.42), 321 (3.62) nm; NMR data see Table S5, and Figure S4; HRESIMS *m/z* 683.2829 [M+H]^+^ (calcd for C_35_H_43_N_2_O_12_, 683.2816) (Table S1).
**Neoantimycin D (5)**. Pale yellow amorphous solid; ${[\alpha ]}_{\text{D}}^{25}$ + 24.1 (*c* = 0.1, CHCl_3_); UV (MeOH) λmax (log ε) 207 (4.10), 227 (3.99), 332 (3.25) nm; NMR data see Table S6, and Figure S5; HR-MS *m/z* 671.3204 [M+H]^+^ (calcd for C_35_H_47_N_2_O_11_, 671.3180) (Table S1).
**Neoantimycin E (6)**. Pale yellow amorphous solid; ${[\alpha ]}_{\text{D}}^{25}$ + 20.1 (*c* = 0.1, CHCl_3_); UV (MeOH) λmax (log ε) 207 (4.09), 228 (4.02), 332 (3.22) nm; NMR data see Table S7, and from Figure S6; HR-MS *m/z* 657.3042 [M+H]^+^ (calcd for C_34_H_45_N_2_O_11_, 657.3023) (Table S1).
**Neoantimycin A (7)**. Pale yellow amorphous solid; ${[\alpha ]}_{\text{D}}^{25}$ + 45.3 (*c* = 0.2, CHCl_3_); UV (MeOH) λmax (log ε) 223 (4.27), 321 (3.49) nm; NMR data see Table S8, and Figure S7; HRESIMS *m/z* 699.3140 [M+H]^+^ (calcd for C_36_H_47_N_2_O_12_, 699.3124) (Table S1).
**Neoantimycin F (8)**. Pale yellow amorphous solid; ${[\alpha ]}_{\text{D}}^{25}$ + 53.7 (*c* = 0.1, CHCl_3_); UV (MeOH) λmax (log ε) 224 (4.42), 321 (3.64) nm; NMR data see Table S9, and Figure S8; HRESIMS *m/z* 685.2983 [M+H]^+^ (calcd for C_35_H_45_N_2_O_12_, 685.2967) (Table S1).

### Preparation of MTPA-Esters of 5-Benzyl-4-Hydroxy-3,3-Dimethyldihydrofuran-2-One

Followed the procedure published by Umeda et al. (2007) 7 (10 mg) was alkaline hydrolyzed to afford 9 (3.4 mg), which was subsequently reacted with either (*S*)- or (*R*)- MTPA-Cl following protocol published by Hoye et al. (2007) to give the corresponding (*R*)- or (*S*)- MTPA-ester (9a and 9b), respectively.

### Preparation of MTPA-Esters of 2-Hydroxy-3-Methylpentanoic Acid

*L*-isoleucine (200 mg) was dissolved in 1 M H_2_SO_4_ (4 mL), an aqueous solution of NaNO_2_ (0.2 g/mL, 4 mL) was slowly added and stirred for 2 h on an ice bath. Then, the reaction mixture was further stirred for 16 h at room temperature. Finally, the reaction mixture was directly extracted with EtOAc (4 mL × 3), The combined organic layer was washed with water (2 × 2 mL) and brine (2 × 2 mL), and dried with Na_2_SO_4_. The organic solvent was removed under reduced pressure and 10 was obtained as colorless oil (136 mg) and subsequently reacted with either (*S*)- or (*R*)- MTPA-Cl following protocol published by Hoye et al. (2007) to give the corresponding (*R*)- or (*S*)- MTPA-ester (10a and 10b), respectively.

### Preparation of MTPA-Esters of 2-Hydroxyisovaleric Acid

(*S*)-2-hydroxyisovaleric acid was purchased from Shanghai yuanye Bio-Technology Co., Ltd, and reacted with either (*S*)- or (*R*)- MTPA-Cl following the protocol published by Hoye et al. (2007) to give the corresponding (*R*)- or (*S*)- MTPA-ester, respectively.

### C_4_ Mosher Analysis of 5-8

Compound 5–8 (100 μg) dissolved in 3 M NaOH (100 μL) were heated to 95°C in a sealed vial for 10 min, after which they were acidified with 6 M HCl (50 μL) and extracted with EtOAc (250 μL). The organic layer was evaporated to dryness at 40°C under a stream of dry N_2_, and the residues were used to prepare (*S*)-MTPA ester derivatives according to the protocol published by Hoye et al. (2007) The (*R*)- or (*S*)- MTPA esters from 5-benzyl-4-hydroxy-3,3-dimethyldihydrofuran-2-one, 2-hydroxy-3-methylpentanoic acid and 2-hydroxyisovaleric acid were adopted as reference substances to determine the absolute configuration of 5, 6, and 8 using LC-MS analysis (Column Phenomenex Jupiter C_4_ column, 5 μm, 150 × 4.6 mm, 1 mL/min, 45% MeCN/H_2_O in 30 min with 0.1% formic acid).

### Marfey's Analysis of 5–8

Compounds 5–8 (50 μg) were dissolved in 300 μL of 6 M HCl and hydrolyzed at 110°C in 3 mL sealed vial for 24 h, whereafter, the HCl was removed by evaporation under the N_2_ gas. The hydrolysate was dissolved in 40 μL of 1 M NaHCO_3_ and treated with 50 μL of Marfey's reagent (1-fluoro-2,4-dinitrophenyl-5-L-alanine amide, FDAA; 1% solution in acetone) at 40°C for 1 h. After that, the mixture was neutralized with 1 M HCl (40 μL), diluted with MeCN (800 μL) and filtered (0.45 μm PTFE) before HPLC-ESIMS analysis (Waters Xbridge C_18_ column, 5 μm, 250 mm × 4.6 mm, 0.8 mL) with a linear gradient elution (10–100% solvent B in 20 min; solvent A: H_2_O with 0.1% formic acid, solvent B: 100% MeCN). Authentic samples of the amino acids *L*-Thr and *L-allo*-Thr were derivatized with *L*-FDAA and *D*-FDAA and analyzed by LC-MS following the same protocol.

### *In vitro* Cytotoxicity Test

A WST-8 colorimetric assay (Cell Counting Kit-8, Dojindo) was used to determine the cytotoxicity of the obtained compounds against four human cancer cell lines (human gastric cancer cell line SGC7901, cisplatin-resistant human gastric cancer cell line SGC7901/DDP, human colon cancer cell line HCT-8, and taxol-resistant human colon cancer cell line HCT-8/T) and noncancerous NCM460 colon cell line. Each cell line (100 μL, 5 × 10^4^ cells/mL) was inoculated into standard 96-well, flat-bottom microplates, and incubated with DMEM (for HCT-8 and NCM460) or RPMI-1640 (for HCT-8/T, SGC7901, and SGC7901/DDP) medium for 24 h at 37°C in a humidified atmosphere containing 5% CO_2_. The attached cells were then incubated with serially diluted NATs (1-8). After continuous exposure to the compounds for 72 h, 10 μL CCK-8 solution was added. After 3 h of incubation at 37°C (5% CO_2_), the absorbance was measured at 450 nm using a microtiter plate reader (SpectraMax 190, Molecular Devices). Cisplatin and taxol (purity ≥ 98% Sigma, USA) were used as positive controls. All studies were performed in triplicate and the IC_50_ values were calculated using Prism software.

### Apoptosis Analysis by Flow Cytometry

An Annexin V-FITC/PI Apoptosis Detection kit I (BD Pharmingen, San Diego, CA, USA) was adopted to examine the cell apoptosis according to the manufacturer's instructions. HCT-8 cells were cultured on a six-well plate (3 × 10^5^ cells/well) and allowed to attach for 24 h before treated with NAT-A (0 and 0.5 μM) for 72 h. Then cells were harvested, washed with PBS and resuspended in 1 × binding buffer. Cell suspension (100 μL) was incubated with 5 μL FITC Annexin-V and 5 μL propidium iodide (PI) for 15 min in the dark. Following the incubation, 400 μL 1 × binding buffer was added into each sample before analysis using flow cytometry (Thermo Fisher Scientific, Eugene, Oregon, USA). The lower left section of fluorocytogram (Annexin V-, PI-) represents the normal cells, lower right section of fluorocytogram (Annexin V+, PI-) represents early apoptosis cells, and upper right section of fluorocytogram (Annexin V+, PI+) represents late apoptosis cells.

### Cell Morphology

HCT-8 cells were seeded in 12-well plates at a density of 1 × 10^5^ cells per well. After overnight adherence, they were exposed to the indicated concentration of NAT-A for 72 h. After that, the cells were washed with PBS and fixed 4% paraformaldehyde for 30 min at room temperature. After washing with PBS, the cells were stained with DAPI (100 ng/mL) for 15 min in the dark and photographed using a fluorescence microscope (Nikon, Japan).

### Statistical Analysis

All the experiments were performed in triplicate, and the values were expressed as the mean ± standard deviation (SD). Data were analyzed using GraphPad Prism 8.0 software (GraphPad Software Inc., La Jolla, CA). Statistical significance was assessed by a two-tailed Student's *t*-test. *P*-values <0.05 were considered statistically significant.

## Result and Discussion

**Figure 1:**
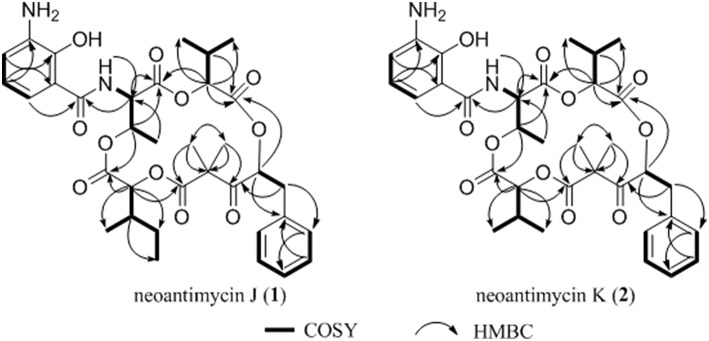
Key ^1^H-^1^H cozy and HMBC correlations for **1–2**.

Compound 1, isolated as a pale yellow amorphous solid, has the molecular formula C_35_H_44_N_2_O_11_ based on HRESIMS data (*m/z* 669.3054, calcd for 669.3023 [M+H]^+^), corresponding to 15 sites of unsaturation. A perusal of the ^1^H and ^13^C NMR (Table 2) revealed that 1 was a depsipeptide with several deshielded methine protons (δ_H_ 4.8–5.7) and 5 putative ester/amid carbonyl carbons (δ_C_ 167–171). Further analyses of ^1^H NMR, ^13^C NMR, ^1^H-^1^H cozy, HMQC, HMBC, and ROESY spectra (Table S2; Figure S1) in DMSO-*d*_6_ suggested that 1 is structurally related to NAT-H (3) with the same characteristic scaffold containing a 4-hydroxy-2,2-dimethyl-3-oxo-5-phenylpentanoic acid moiety, a threonine, and two aliphatic hydroxycarboxylic acid residues. However, the obvious ^13^C chemical shift differences of aromatic carbons (δ_C−26_ 117.6 and δ_C−28_ 115.5), as well as the absence of formyl signal (δ_C_ 160.4) suggested that the *N*-formyl aminosalicylic acid unit in 3 was replaced by 3-amino-2-hydroxybenzoic acid moiety in 1. Assembly of these residues was assisted by diagnostic COSY and HMBC correlations (Figure 1).

**Table 2:** ^1^H (600 MHz) and ^13^C (150 MHz) NMR Data for 1 and 2 in DMSO-*d*_6_.

	**1**		**2**	
**No**	**δ_H_, mult. (*J* in Hz)**	**δ_C_**	**δ_H_, mult. (*J* in Hz)**	**δ_C_**
1		202.6		202.6
2	5.68, *t* (6.7)	76.5	5.69, dd (7.8, 5.6)	76.5
3		167.2		167.2
4	5.05, overlapped	76.2	5.06, d (5.3)	76.2
5		168.0^a^		167.9^a^
6	5.05, overlapped	55.6	5.03, br d (8.3)	55.7
7	5.58, dt (6.0, 2.8)	70.5	5.58, dt (7.0, 4.9)	70.5
8		167.7^a^		167.6^a^
9	4.86, br d (7.7)	75.7	4.80, d (7.3)	76.8
10		171.1		171.1
11		54.3		54.3
12	3.15, dd (14.1, 5.6)	37.2	3.15, dd (14.0, 5.5)	37.2
	3.06, dd (14.0, 7.9)		3.06, dd (14.0, 7.8)	
13		135.5		135.4
14/18	7.20, d (7.3)	129.6	7.20, d (7.4)	129.6
15/17	7.30, *t* (7.4)	128.4	7.30, *t* (7.4)	128.4
16	7.24, m	127.0	7.24, d (7.3)	127.0
19	2.01, m	30.1	2.01, m	30.1
20	0.68, d (6.8)	16.8	0.68, d (6.9)	16.8
21	0.82, overlapped	17.9	0.82, d (6.9)	17.9
22		170.9		170.8
23		113.7		113.7
24		147.7		147.9
25		137.7		137.2
26	6.82, d (7.7)	117.6	6.85, d (7.4)	118.0
27	6.68, *t* (7.9)	118.6	6.69, *t* (7.9)	118.6
28	7.35, d (8.0)	115.5	7.38, dd (8.2, 3.9)	115.9
29	1.23, d (6.2)	15.7	1.23, d (6.4)	15.7
30	1.88, m	35.8	2.06, m	29.9
31	0.86, d (6.8)	14.1	0.88, d (6.8)	17.6^a^
32	1.43, m	24.0	0.88, d (6.8)	17.7^a^
	1.13, m			
33	1.34, s	21.3	1.34, s	21.3
34	1.21, s	21.2	1.21, s	21.2
35	0.82, overlapped	10.5		
6-NH	8.93, br d (8.2)		8.93, br d (8.2)	

a *values are interchangeable*.

The deduction of compound 2 followed a similar workflow. The molecular formula was elucidated as C_34_H_42_N_2_O_11_ based on HRESIMS analysis as well as its ^1^H-NMR and ^13^C NMR data (Table 2), which suggested that 2 had one CH_2_ <1. Further analysis of the ^1^H-^1^H COSY spectrum revealed two spin systems of OH-CH-CH-(CH_3_)_2_, indicating that the 2-hydroxy-3-methyl valerate in 1 was replaced by the 2-hydroxyisovalerate unit in 2. The connection of each unit was also confirmed by ^1^H-^1^H COSY and HMBC (Figure 1). Both 1 and 2 are new compounds and named neoantimycin J and K, respectively.

The molecular formula of 3 was determined to be C_36_H_44_N_2_O_12_ and confirmed to be neoantimycin H by a comparison of NMR and OR data with those previously reported (Lee, 2014) (Table S4). The molecular formula of 4 was elucidated as C_35_H_42_N_2_O_12_ and designated as neoantimycin I (Table S5) without a full stereochemistry assignment in our last study (Zhou et al., 2018).

**Scheme 1:**
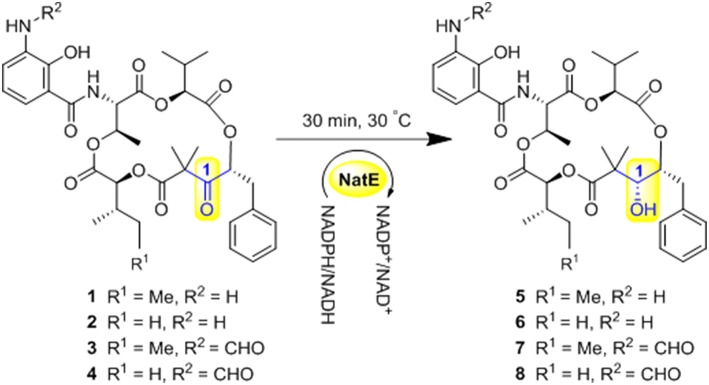
Ketoreductase NatE catalyze the reduction of **1–4** to generate **5–8**.

**Figure 2:**
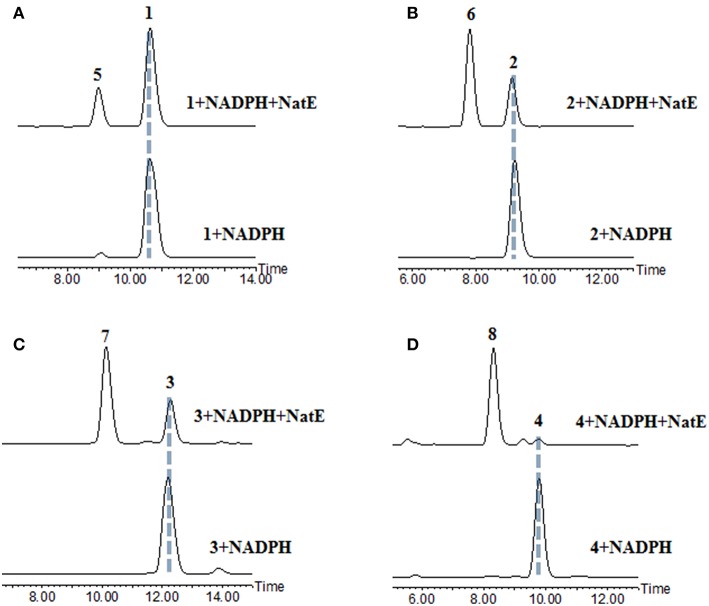
HPLC analysis of the products generated in the NatE enzymatic conversion. The LC-MS data are displayed with total ion current. **(A)** 1 was converted to 5. **(B)** 2 was converted to 6. **(C)** 3 was converted to 7. **(D)** 4 was converted to 8.

NatE is a ketoreductase reported in our previous study (Zhou et al., 2018), which could convert the oxidized type of NATs to their corresponding reduced products at C-1 specifically *in vitro and in vivo*. To confirm the absolute configuration of 1, 2, and 4, we applied NatE to generate 5, 6, and 8, respectively, as well as 7 from 3 to facilitate stereochemistry assignment. Compounds 1–4 were incubated with the recombinant NatE and NADPH or NADH, the corresponding reduced products 5–8 were generated as observed in the HPLC-MS analysis (Scheme 1; Figure 2), and then be purified using the semi-preparative HPLC method. The molecular formula of 7 was determined to be C_36_H_46_N_2_O_12_ based on HRESIMS analysis, and it was further confirmed to be NAT-A in our later experiment and by comparison of OR and NMR data (Figure S7; Table S8) with those previously reported (Salim et al., 2014). Analogously, 5, 6, and 8 converted from 1, 2, and 4 were deduced as NAT-D, E, and F, respectively, in the planar structures (Li et al., 2013), which were also found in the wild type of *Streptomyces conglobatus*. In addition, based on the HMBC correlations in our experiment and the spectroscopic data of JBIR-04 (Izumikawa et al., 2007), the chemical shifts of 5 at 14.4 ppm and 0.88 ppm were ascribed to C-31 and H-31, respectively, which is different from those at Li's report (C-31 at 21.9 ppm and H-31 at 1.33 ppm) (Li et al., 2013).

**Figure 3:**
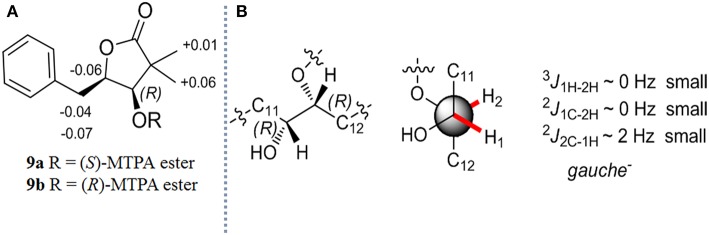
**(A)** Δδ (δ_*S*_ - δ_*R*_) values obtained from the MTPA esters of **9 (B)** The absolute stereochemistry of C-1 and C-2 in **7**.

After the reduced derivatives of 1–4 were generated, the configurational assignment could be completed according to the reported methods for NATs with C1-hydroxyl (Umeda et al., 2007; Salim et al., 2014). The stereochemistry of 7 was reconfirmed for proving the feasibility of our method. The absolute configuration at C-1 was determined by the modified Mosher's method (Hoye et al., 2007). Alkaline hydrolysis of 7 (10 mg) yields 5-benzyl-4-hydroxy-3,3-dimethyldihydrofuran-2-one (9), which was subsequently obtained from the ether layer and converted to (*S*)- and (*R*)-MTPA esters (9a and 9b) by reacted with (*R*)- and (*S*)-MTPA-Cl, respectively. (Table S10; Figure S9) The distribution of the values of the Δδ_H_ values (Δδ_H_ = δ_S_ - δ_R_) allowed us to assign the *R* configuration at the C-1 position (Figure 3A). Compounds 9a and 9b were hereinafter adopted as reference substances (Figure S11A) to ascertain the absolute configuration of C-1 in other compounds.

The stereochemistry at C-2 in 7 was confirmed by the determination of the relative configurations between C-1 and C-2 employing the HETLOC method (Kurz et al., 1991). The vicinal coupling constant value measured from HETLOC assay (Figure S5C) for ^3^*J*_1H−2H_ (~0 Hz) as well as the small heteronuclear long-range coupling constants (^2^*J*_1C−2H_ ~ 0 Hz and ^2^*J*_2C−1H_ ~ 2 Hz) suggested a *gauche* disposition between H-1 and H-2, while H-1 and an oxygen atom at C-2, and H-2 and an oxygen atom at C-1 are both in the anti-position as is depicted in Figure 3B, which established the *R* configuration at C-2.

**Figure 4:**
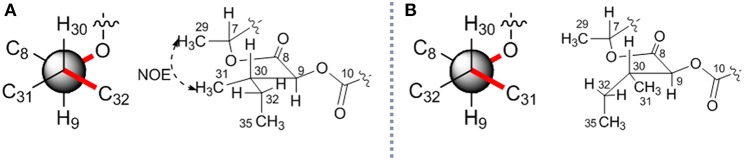
The relative stereochemistry of C-9 and C-30 in **7**. Two possible relative structures **(A,B)**.

The stereochemistry of C-9 and C-30 can be determined by using *J* coupling analysis combined with NOE analysis (Umeda et al., 2007). A large ^3^*J*_9H−30H_ coupling constant (8.3 Hz), as well as two small coupling constants (^3^J_9H−31C_ ~ 2 Hz and ^3^J_9H−32C_ ~ 0 Hz), suggested that 9-H and 30-H protons were in the *anti* orientation. An NOE (Figure 4A) between H-29 (δ_H_ 1.34) and H-31 (δ_H_ 0.88) constructed two possible stereochemistry at 2-hydroxy-3-methylpentanoic acid residues as 2*S*, 3*S* or 2*R*, 3*R*.

To assign the absolute configurations, analytical chromatography was applied (Salim et al., 2014). The authentic sample of *L*-isoleucic acid (10) (Figure S10) was initially synthesized from *L*-isoleucine as previously reported (Banasik et al., 2016), then derivatized with the (*S*)- and (*R*)-MTPA-Cl to yield authentic standards of the (*R*)- and (*S*)-MTPA esters (10a and 10b), the latter being chromatographic surrogate for the (*R*)-MTPA esters of *D*-isoleucic acid. Meanwhile, the alkaline hydrolysate of 7 was reacted with (*R*)-MTPA-Cl and subsequently dissolved in MeOH. Both of the standard and sample solution were subjected to C_4_ Mosher analysis. According to the retention time in LC-MS (Figure S11B), compound 7 incorporated (2*S*, 3*S*)-2-hydroxy-3-methylpentanoic acid residue. Conclusively, the stereochemistry at the C-9 and C-30 position in 7 was established as 9*S*, 30*S*.

The absolute configuration of C-4 was also determined by analytical chromatography. The standard of (*S*)-2-hydroxyisovaleric acid (11) was reacted with the *S*- and *R*-MTPA-Cl to yield authentic standards of the *R*- and *S*-MTPA esters (11a and 11b), which were then subjected to C_4_ Mosher analysis. By comparison of the retention time in LC-MS (Figure S11B), the stereochemistry at the C-4 was established as *S*. The stereochemistry at C-6 and C-7 was assigned by advanced Marfey's analysis. *L*-threonine and *L*-allo-threonine were reacted with *L*- and *D*-FDLA (1-fluoro-2,4-dinitrophenyl-5-leucinamide), respectively. Acid hydrolysate of 7 was reacted with *L*-FDLA, and eluted as a single peak at the same position compared with that of the *L*-FDLA *L*-Threonine conjugates, indicating the presence of *L*-threonine (Figure S12). Conclusively, the absolute stereochemistry of 7 was determined as 1*R*, 2*R*, 4*S*, 6*S*, 7*R*, 9*S*, 30*S*, and 3 as 2*R*, 4*S*, 6*S*, 7*R*, 9*S*, 30*S*, correspondingly.

The configurational assignment of other compounds was completed under the following procedure. Advanced Marfey's analysis of acid hydrolysates confirmed the existence of *L*-threonine residue (Figure S12). (*S*)-2-hydroxyisovaleric acid, (2*S*, 3*S*)-2-hydroxy-3-methylvaleric acid, and (3*R*, 4*R*)-3,4-dihydroxy-2,2-dimethyl-5-phenylvaleric acid residues were also confirmed by comprehensive analysis of ^3^*J*_HH_ coupling constants, *J*-based configurational spectroscopy, NOE analysis, and LC-MS chromatography Finally, the absolute stereochemistry of 1 was determined as 2*R*, 4*S*, 6*S*, 7*R*, 9*S*, 30*S*, 2 and 4 as 2*R*, 4*S*, 6*S*, 7*R*, 9*S*, 5 as 1*R*, 2*R*, 4*S*, 6*S*, 7*R*, 9*S*, 30*S*, 6, and 8 as 1*R*, 2*R*, 4*S*, 6*S*, 7*R*, 9*S*.

Compounds 3, 4, 7, and 8 showed outstanding and broad-spectrum anti-cancer activities in our previous work (Zhou et al., 2018), which encourage us to further analyze the structure-activity relationship (SAR) of these 15-membered depsipeptides, especially toward the drug-resistant cancer cells. We examined the cytotoxicity of 1–8 against two sets of drug-sensitive and resistant human cancer cell lines (Figures S13–S16): the human gastric cancer cell line SGC7901 and its resistant counterpart SGC7901/DDP (resistant to cisplatin), the human colon cancer cell line HCT-8 and its resistant counterpart HTC-8/T (resistant to taxol). As shown in Table 3, compounds 3, 4, 7, 8 exhibited significant anti-proliferative activities against all four cell lines (1.5~522.7 nM), stronger than cisplatin and taxol. Of note, these four compounds displayed extraordinarily excellent anti-proliferative activity (5.2~33.5-fold) in the drug-resistant cell lines than in the sensitive ones, demonstrating their potentiality to overcome the drug resistance frequently occurring in clinical tumor therapy. The comparison of the relative activity levels of 1 vs. 3, 2 vs. 4, 5 vs. 7, and 6 vs. 8 demonstrated that the *N*-formyl group at aminosalicylic acid moiety is indeed a sensitive site: removal of formamide group almost deprived NATs of their excellent cytotoxicity. Compounds 7 and 8 displayed approximately 1.2~13-fold higher activity *in vitro* than their corresponding oxidized forms (3 and 4), indicating that the hydroxyl at C-1 position contributes to the anti-cancer activity considerably. In addition, when R^1^ was substituted by a methyl group, the cytotoxic activities were enhanced observably, especially for the activities against the drug-resistant cancer cells between 7 and 8, 2~13-fold increment hinting at the involvement of the alkyl substitution in the cytotoxicity. Subsequently, the noncancerous NCM460 colon cell was used to detect the toxic selectivities of compound 3, 4, 7, and 8 since they exhibited potent antiproliferation against the cancer cells. Interestingly, we found that compound 4, 7, and 8 had little toxic effect with IC_50_ values > 50 μM, while 3 showed considerable toxicity with IC_50_ at 114.8 ± 18.7 nM, we suspected that the concurrence of keto group at C-1 and the alkyl extension at C-32 could affect the selective cytotoxicity of NATs, and further investigation would be carried out if more NATs containing C-1 keto and C-32 alkyl extension were obtained in future.

**Table 3:** Cytotoxicity of **1**–**8** against Four Cancer Cell Lines and A Noncancerous Cell Line *in vitro* (*n* = 3).

	**IC_50_ (nM)**				
	**SGC7901**	**SGC7901/DDP**	**HCT-8**	**HCT-8/T**	**NCM460**
1	>20,000	>20,000	>20,000	>20,000	n/a
2	>20,000	>20,000	>20,000	>20,000	n/a
3	103.5 ± 22.7	12.2 ± 4.6	522.7 ± 103.6	26.6 ± 1.5	114.8 ± 18.7
4	179.4 ± 18.3	15.7 ± 1.6	232.7 ± 20.1	32.6 ± 4.2	>50,000
5	10,167.7 ± 4,088.3	>20,000	>20,000	>20,000	n/a
6	1,774.7 ± 144.0	>20,000	13,120.6	>20,000	n/a
7	24.5 ± 3.3	1.5 ± 0.1	66.9 ± 5.7	2.0 ± 0.1	>50,000
8	98.5 ± 32.6	6.4 ± 0.5	136.2 ± 18.2	26.1 ± 0.1	>50,000
cisplatin^a^	2,559.4 ± 247.8	5,673.1 ± 336.7	9,494.9 ± 1,161.3	>20,000	n/a
taxol^a^	n/a	n/a	2,517.1 ± 368.2	>20,000	n/a

a cisplatin and taxol served as positive controls.

*Values shown are means ± SD of triplicate determination*.

Apoptosis is a common type of cell death. To investigate whether the cell apoptosis was involved in cell viability reduction, the HCT-8 cells treated with different concentrations of compound 7 for 72 h were analyzed by flow cytometry. After being stained with Annexin V/PI, the 7 administration group led to the increased percentage (from 7.2 ± 1.1 to 25.9 ± 0.7%) of apoptotic cells (Figures S17A,B). Moreover, 4,6-diamidino-2-phenylindole dihydrochloride (DAPI) staining was also used. As shown in Figure S17C, the untreated cells were stained equably with blue fluorescence under the fluorescent microscope, demonstrating the steady chromatinic distribution in the nucleolus. The HCT-8 cells treated with 7 exhibited nuclear shrinkage, DNA fragments as well as chromatin condensation. Taken together, these results indicated that NATs have the ability to inhibit cell growth and induce typical apoptosis in human colon cancer cells.

In conclusion, as part of our extended research for new NATs, two new 15-membered depsipeptides, NAT-J and K (1 and 2) were isolated, which expands the members of the NATs family. An Integrated methodology of enzymatic conversion, spectroscopic method, chemical derivatization, and chromatography was developed to assign/reconfirm the absolute configuration for the new as well as the known neoantimycins. Three NATs (4, 7, and 8) in the work displayed outstanding inhibition activities toward gastric or colon drug-resistant cancer cells but did not exhibit cytotoxicity toward the noncancerous NCM460 colon cell, suggesting that they could be potential antitumor candidates for further development. The SAR study indicated that the hydroxyl at the C-1 position, a longer alkyl substitution at C-32, and the *N*-formyl residues on the side benzene ring play a key role in enhancing the anticancer effect. Though the two new compounds (1 and 2) had no significant cytotoxicity compared with the known NATs (3, 4, 7, and 8), the SAR investigation in our study indicated that the active pharmacophore of NATs and should shed light on further optimization by bioengineering or chemical synthesis. In addition, it was also found that NATs could induce the apoptosis of tumor cells. The pharmacological study of NATs is still ongoing.

## Data Availability

The raw data supporting the conclusions of this manuscript will be made available by the authors, without undue reservation, to any qualified researcher.

## Author Contributions

XL, LL, HZ, YC, SW, FS, LC, and BL performed the experiments. XL identified the structures. YZ, SX, and H-WL conceived and designed the experiments. XL and YZ wrote the paper. All authors have approved the final version of the manuscript.

### Conflict of Interest Statement

The authors declare that the research was conducted in the absence of any commercial or financial relationships that could be construed as a potential conflict of interest.

## Supplementary Material

These data include general experimental procedures, HRESIMS, 1D and 2D NMR, Advanced Marfey's and C_4_ Mosher analysis, and CCK-8 cytotoxicity assays.
