# Supplementary material for: Compound Discovery and Structure-Activity Relationship Study of Neoantimycins Against Drug-Resistant Cancer Cells
Source: Front Chem. 2019 Jul 5;7:481. doi: 10.3389/fchem.2019.00481 (PMC6624652; doi:10.3389/fchem.2019.00481)
Supplement: Supplementary file 1 [file Data_Sheet_1.docx]

***Supplementary Material***

**Table of Contents**

[Table S1. HRESIMS data of the compounds 1-8 3](#_Toc11149790)

[Table S2. NMR (600 MHz, DMSO-*d*_6_) data for 1 4](#_Toc11149791)

[Table S3. NMR (600 MHz, DMSO-*d*_6_) data for 2 5](#_Toc11149792)

[Table S4. NMR (600 MHz, DMSO-*d*_6_) data for 3 6](#_Toc11149793)

[Table S5. NMR (600 MHz, DMSO-*d*_6_) data for 4 8](#_Toc11149794)

[Table S6. NMR (600 MHz, CDCl_3_) data for 5 9](#_Toc11149795)

[Table S7. NMR (600 MHz, CDCl_3_) data for 6 11](#_Toc11149796)

[Table S8. NMR (600 MHz, CDCl_3_) data for 7 13](#_Toc11149797)

[Table S9. NMR (600 MHz, CDCl_3_) data for 8 15](#_Toc11149798)

[Table 10. ^1^H NMR (600 MHz CDCl_3_) Data for the (*S*)- and (*R*)-MTPA Esters of 9 17](#_Toc11149799)

[Figure S1a ^1^H NMR spectrum of 1 in DMSO-*d*_6_ 18](#_Toc11149800)

[Figure S1b ^13^C NMR spectrum of 1 in DMSO-*d*_6_ 19](#_Toc11149801)

[Figure S1c HSQC spectrum of 1 in DMSO-*d*_6_ 20](#_Toc11149802)

[Figure S1d HMBC spectrum of 1 in DMSO-*d*_6_ 21](#_Toc11149803)

[Figure S1e ^1^H-^1^H COSY spectrum of 1 in DMSO-*d*_6_ 22](#_Toc11149804)

[Figure S1f ROESY spectrum of 1 in DMSO-*d*_6_ 23](#_Toc11149805)

[Figure S2a ^1^H NMR spectrum of 2 in DMSO-*d*_6_ 24](#_Toc11149806)

[Figure S2b ^13^C NMR spectrum of 2 in DMSO-*d*_6_ 25](#_Toc11149807)

[Figure S2c HSQC spectrum of 2 in DMSO-*d*_6_ 26](#_Toc11149808)

[Figure S2d HMBC spectrum of 2 in DMSO-*d*_6_ 27](#_Toc11149809)

[Figure S2e ^1^H-^1^H COSY spectrum of 2 in DMSO-*d*_6_ 28](#_Toc11149810)

[Figure S2f ROESY spectrum of 2 in DMSO-*d*_6_ 29](#_Toc11149811)

[Figure S3a ^1^H NMR spectrum of 3 in DMSO-*d*_6_ 30](#_Toc11149812)

[Figure S3b ^13^C NMR spectrum of 3 in DMSO-*d*_6_ 31](#_Toc11149813)

[Figure S4a ^1^H NMR spectrum of 4 in DMSO-*d*_6_ 32](#_Toc11149814)

[Figure S4b ^13^C NMR spectrum of 4 in DMSO-*d*_6_ 33](#_Toc11149815)

[Figure S5a ^1^H NMR spectrum of 5 in CDCl_3_ 34](#_Toc11149816)

[Figure S5b ^13^C NMR spectrum of 5 in CDCl_3_ 35](#_Toc11149817)

[Figure S5c HETLOC spectrum of 5 in CDCl_3_ 36](#_Toc11149818)

[Figure S6a ^1^H NMR spectrum of 6 in CDCl_3_ 37](#_Toc11149819)

[Figure S6b ^13^C NMR spectrum of 6 in CDCl_3_ 38](#_Toc11149820)

[Figure S6c HETLOC spectrum of 6 in CDCl_3_ 39](#_Toc11149821)

[Figure S7a ^1^H NMR spectrum of 7 in CDCl_3_ 40](#_Toc11149822)

[Figure S7b ^13^C NMR spectrum of 7 in CDCl_3_ 41](#_Toc11149823)

[Figure S7c HETLOC spectrum of 7 in CDCl_3_ 42](#_Toc11149824)

[Figure S8a ^1^H NMR spectrum of 8 in CDCl_3_ 43](#_Toc11149825)

[Figure S8b ^13^C NMR spectrum of 8 in CDCl_3_ 44](#_Toc11149826)

[Figure S8c HETLOC spectrum of 8 in CDCl_3_ 45](#_Toc11149827)

[Figure S9a ^1^H NMR spectrum of 9 in CDCl_3_ 46](#_Toc11149828)

[Figure S9b ^1^H NMR spectrum of *S*-MTPA ester of 9 in CDCl_3_ 47](#_Toc11149829)

[Figure S9c ^1^H NMR spectrum of *R*-MTPA ester of 9 in CDCl_3_ 48](#_Toc11149830)

[Figure S10a ^1^H NMR spectrum of 10 in CDCl_3_ 49](#_Toc11149831)

[Figure S10b ^13^C NMR spectrum of 10 in CDCl_3_ 50](#_Toc11149832)

[Figure S11a. LCMS chromatogram of extracted ion at 437 [M + H]^+^from Mosher’s esterification reaction 51](#_Toc11149833)

[Figure S11b. LC-MS chromatogram of extracted ion at 333 [M - H] and 347 [M - H]^-^ from Mosher’s esterification reaction 52](#_Toc11149834)

[Figure S12. LC-MS chromatogram of extracted ion at 414 [M + H]^+^ from FDLA derivatization reaction 53](#_Toc11149835)

[Figure S13. Graphs for CCK-8 cytotoxicity assays in HCT-8/T cell line 54](#_Toc11149836)

[Figure S14. Graphs for CCK-8 cytotoxicity assays in HCT-8 cell line 55](#_Toc11149837)

[Figure S15. Graphs for CCK-8 cytotoxicity assays in SGC7901/DDP cell line 56](#_Toc11149838)

[Figure S16. Graphs for CCK-8 cytotoxicity assays in SGC7901 cell line 57](#_Toc11149839)

[Figure S17. Effects of NAT-A (3) on cell proliferation and apoptosis in HCT-8 cells. 58](#_Toc11149840)

## Table S1. HRESIMS data of the compounds 1-8

| **Compounds** | **formula** | **det. *m/z*** | **calc. *m/z*** | **Error (ppm)** |
| --- | --- | --- | --- | --- |
| **1** | H^+^C_35_H_44_N_2_O_11_ | 669.3054 | 669.3023 | 4.63 |
| **2** | H^+^C_34_H_42_N_2_O_11_ | 655.2896 | 655.2867 | 4.43 |
| **3** | H^+^C_36_H_44_N_2_O_12_ | 697.2987 | 697.2972 | 2.15 |
| **4** | H^+^C_35_H_42_N_2_O_12_ | 683.2829 | 683.2816 | 1.90 |
| **5** | H^+^C_35_H_46_N_2_O_11_ | 671.3204 | 671.3180 | 3.58 |
| **6** | H^+^C_34_H_44_N_2_O_11_ | 657.3042 | 657.3023 | 2.89 |
| **7** | H^+^C_36_H_46_N_2_O_12_ | 699.3140 | 699.3124 | 2.29 |
| **8** | H^+^C_35_H_44_N_2_O_12_ | 685.2983 | 685.2967 | 2.33 |

## Table S2. NMR (600 MHz, DMSO-*d*_6_) data for 1

|  | δ_C_ | δ_H_ | HMBC | COSY | ROESY |
| --- | --- | --- | --- | --- | --- |
| 1 | 202.6 |  |  |  |  |
| 2 | 76.5 | 5.68, t (6.7) | 1, 3, 12, 13 | 12a, 12b | 14/18, 33 |
| 3 | 167.2 |  |  |  |  |
| 4 | 76.2 | 5.05, overlap | 3, 5, 19, 20, 21 | 19 | 20, 21 |
| 5 | 168.0^a^ |  |  |  |  |
| 6 | 55.6 | 5.05, overlap | 5 | 7, 6-NH | 29 |
| 7 | 70.5 | 5.58, dt (6.0, 2.8) | 5, 8 | 6, 29 | 6-NH |
| 8 | 167.7^a^ |  |  |  |  |
| 9 | 75.7 | 4.86, br d (7.7) | 8, 10, 30, 31, 32 | 30 | 31, 32 |
| 10 | 171.1 |  |  |  |  |
| 11 | 54.3 |  |  |  |  |
| 12 | 37.2 | 3.15, dd (14.1, 5.6) | 1, 2, 13, 14/18 | 2 | 33 |
|  |  | 3.06, dd (14.0, 7.9) |  |  |  |
| 13 | 135.5 |  |  |  |  |
| 14/18 | 129.6 | 7.20, d (7.3) | 12, 16 | 15/17 | 2, 12 |
| 15/17 | 128.4 | 7.30, t (7.4) | 13 | 14/18, 16 |  |
| 16 | 127.0 | 7.24, m | 14/18 | 15/17 |  |
| 19 | 30.1 | 2.01, m | 3, 4, 20, 21 | 4, 20, 21 |  |
| 20 | 16.8 | 0.68, d (6.8) | 4, 19, 21 | 19 | 4 |
| 21 | 17.9 | 0.82, ovelap | 4, 19, 20 | 19 | 4 |
| 22 | 170.9 |  |  |  |  |
| 23 | 113.7 |  |  |  |  |
| 24 | 147.7 |  |  |  |  |
| 25 | 137.7 |  |  |  |  |
| 26 | 117.6 | 6.82, d (7.7) | 24, 28 | 27 |  |
| 27 | 118.6 | 6.68, t (7.9) | 23, 25, 26 | 26, 28 |  |
| 28 | 115.5 | 7.35, d (8.0) | 22, 24, 26 | 27 |  |
| 29 | 15.7 | 1.23, d (6.2) | 6, 7 | 7 | 6 |
| 30 | 35.8 | 1.88, m |  | 31, 32 |  |
| 31 | 14.1 | 0.86, d (6.8) | 9, 30, 32 | 30 |  |
| 32 | 24.0 | 1.43, m | 31, 35 | 30, 35 |  |
|  |  | 1.13, m | 9, 30, 31, 35 |  |  |
| 33 | 21.3 | 1.34, s | 1, 10, 11, 34 |  | 2 |
| 34 | 21.2 | 1.21, s | 1, 10, 11, 33 |  |  |
| 35 | 10.5 | 0.82, overlap | 30, 32 | 32 |  |
| 6-NH |  | 8.93, br d (8.2) | 22 | 6 |  |

^a^ values are interchangeable

## Table S3. NMR (600 MHz, DMSO-*d*_6_) data for 2

|  | δ_C_ | δ_H_ | HMBC | COSY | ROESY |
| --- | --- | --- | --- | --- | --- |
| 1 | 202.6 |  |  |  |  |
| 2 | 76.5 | 5.69, dd (7.8, 5.6) | 1, 3, 12, 13 | 12a, 12b | 14/18, 33 |
| 3 | 167.2 |  |  |  |  |
| 4 | 76.2 | 5.06, d (5.3) | 3, 5, 19, 20, 21 | 19 | 20, 21 |
| 5 | 167.9^a^ |  |  |  |  |
| 6 | 55.7 | 5.03, br d (8.3) | 5, 22 | 7, 6-NH | 29 |
| 7 | 70.5 | 5.58, dt (7.0, 4.9) | 5, 8 | 6, 29 | 6-NH |
| 8 | 167.6^a^ |  |  |  |  |
| 9 | 76.8 | 4.80, d (7.3) | 8, 10, 30, 31, 32 | 30 | 7, 31, 32, 6-NH |
| 10 | 171.1 |  |  |  |  |
| 11 | 54.3 |  |  |  |  |
| 12 | 37.2 | 3.15, dd (14.0, 5.5) | 1, 2, 13, 14/18 | 2 | 33, 34 |
|  |  | 3.06, dd (14.0, 7.8) |  |  | 34 |
| 13 | 135.4 |  |  |  |  |
| 14/18 | 129.6 | 7.20, d (7.4) | 12, 16 | 15/17 | 2, 12 |
| 15/17 | 128.4 | 7.30, t (7.4) | 13 | 14/18, 16 |  |
| 16 | 127.0 | 7.24, d (7.3) | 14/18 | 15/17 |  |
| 19 | 30.1 | 2.01, m | 3, 4, 20, 21 | 4, 20, 21 |  |
| 20 | 16.8 | 0.68, d (6.9) | 4, 19, 21 | 19 |  |
| 21 | 17.9 | 0.82, d (6.9) | 4, 19, 20 | 19 |  |
| 22 | 170.8 |  |  |  |  |
| 23 | 113.7 |  |  |  |  |
| 24 | 147.9 |  |  |  |  |
| 25 | 137.2 |  |  |  |  |
| 26 | 118.0 | 6.85, d (7.4) | 24, 28 | 27 |  |
| 27 | 118.6 | 6.69, t (7.9) | 23, 25, 26 | 26, 28 |  |
| 28 | 115.9 | 7.38, dd (8.2, 3.9) | 22, 24, 26 | 27 |  |
| 29 | 15.7 | 1.23, d (6.4) | 6, 7 | 7 | 6 |
| 30 | 29.9 | 2.06, m | 8, 9, 31, 32 | 31, 32 |  |
| 31 | 17.6^a^ | 0.88, d (6.8) | 9, 30, 32 | 30 |  |
| 32 | 17.7^a^ | 0.88, d (6.8) | 9, 30, 31 | 30 |  |
| 33 | 21.3 | 1.34, s | 1, 10, 11, 34 |  | 2 |
| 34 | 21.2 | 1.21, s | 1, 10, 11, 33 |  |  |
| 6-NH |  | 8.93, br d (8.2) | 22 | 6 |  |

^a^ values are interchangeable

## Table S4. NMR (600 MHz, DMSO-*d*_6_) data for 3

|  | Experimental *δ*_C_ (125 MHz) | Literature^a^ *δ*_C_ (125 MHz) | Experimental *δ*_H_ (600 MHz) | Literature^a^  *δ*_H_ (600 MHz) |
| --- | --- | --- | --- | --- |
| 1 | 202.4 | 202.4 |  |  |
| 2 | 76.4 | 76.5 | 5.69, dd (7.8, 5.6) | 5.38, dd (10.0,2.7) |
| 3 | 167.1 | 168.1 |  |  |
| 4 | 76.2 | 75.7 | 5.08, d (5.2) | 5.04, d (2.9) |
| 5 | 167.6^b^ | 168.1 |  |  |
| 6 | 55.7 | 55.1 | 5.10, dd (8.2, 3.1) | 5.27, dd (8.8, 2.9) |
| 7 | 70.4 | 70.0 | 5.62, dd (6.5, 3.2) | 5.70, qd (6.4, 2.9) |
| 8 | 167.7^b^ | 167.6 |  |  |
| 9 | 75.6 | 75.5 | 4.87, d (7.8) | 5.24, d (7.9) |
| 10 | 171.1 | 170.8 |  |  |
| 11 | 54.2 | 55.1 |  |  |
| 12 | 37.1 | 36.5 | 3.16, dd (14.1, 5.6) | 3.15, dd (14.7, 2.7) |
|  |  |  | 3.07, dd (14.1, 7.9) | 2.91, dd (14.7, 10.0) |
| 13 | 135.4 | 136.2 |  |  |
| 14/18 | 129.6 | 129.2 | 7.20, d (7.2) | 7.27, d (7.3) |
| 15/17 | 128.4 | 128.5 | 7.30, t (7.4) | 7.32, dd (7.3, 7.3) |
| 16 | 126.9 | 126.9 | 7.24, t (7.2) | 7.24, t (7.3) |
| 19 | 30.1 | 29.7 | 2.02, m | 1.99, qqd (6.9, 6.9, 2.9) |
| 20 | 16.7 | 16.1 | 0.69, d (6.8) | 0.79, d (6.9) |
| 21 | 17.8 | 18.2 | 0.83, overlap | 0.91, d (6.9) |
| 22 | 170.3 | 170.1 |  |  |
| 23 | 114.4 | not listed^c^ |  |  |
| 24 | 150.4 | 150.6 |  |  |
| 25 | 126.9 | 126.9 |  |  |
| 26 | 125.1 | 125.1 | 8.25, dd (7.9, 1.5) | 8.21, d (7.4) |
| 27 | 118.2 | 118.1 | 6.95, t (8.0) | 6.93, br s |
| 28 | 123.5 | 123.6 | 7.95, dd (8.2, 1.6) | 7.95, br s |
| 29 | 15.6 | 16.1 | 1.25, d (6.5) | 1.21, d (6.5) |
| 30 | 35.7 | 36.8 | 1.89, m | 1.82, dqd (7.9, 6.8, 4.1) |
| 31 | 14.0 | 13.8 | 0.87 d (6.9) | 0.87, d (6.8) |
| 32 | 24.0 | 24.1 | 1.44, m | 1.45, dqd (14.3, 7.2, 4.1) |
|  |  |  | 1.14, m | 1.08, dq (14.3, 7.2) |
| 33 | 21.2 | 20.6 | 1.34, s | 1.42, s |
| 34 | 21.2 | 21.2 | 1.21, s | 1.32, s |
| 35 | 10.4 | 10.6 | 0.83, overlap | 0.88, dd (7.2, 7.2) |
| CHO | 160.4 | 160.4 | 8.34, d (1.9) | 8.31, d (1.8) |
| 6-NH |  |  | 9.21, d (8.0) | 9.27, br s |
| 24-OH |  |  | 12.72, br s | 12.8, br s |
| 25-NH |  |  | 9.83, d (2.1) | 9.82, br s |

^a^ Salim, A. A. et al. Org. Lett. 16, 5036–5039 (2014)

^b^ values are interchangeable

^c^ Value was not listed in the literature value but was observed in the spectrum acquired in our laboratory

## Table S5. NMR (600 MHz, DMSO-*d*_6_) data for 4

|  | δ_C_ | δ_H_ | HMBC | COSY | ROESY |
| --- | --- | --- | --- | --- | --- |
| 1 | 202.5 |  |  |  |  |
| 2 | 76.5 | 5.70, dd (7.8, 5.6) | 1, 3, 12, 13 | 12a, 12b | 33 |
| 3 | 167.1 |  |  |  |  |
| 4 | 76.2 | 5.08, d (6.7) | 3, 19, 20, 21 | 19 | 20, 21 |
| 5 | 167.7 |  |  |  |  |
| 6 | 55.7 | 5.09, m | 5 | 7, 6-NH | 29 |
| 7 | 70.4 | 5.61, m | 5, 29 | 6, 29 |  |
| 8 | 167.5 |  |  |  |  |
| 9 | 76.7 | 4.82, d (7.3) | 8, 10, 30, 31, 32 | 30 | 31, 32 |
| 10 | 171.1 |  |  |  |  |
| 11 | 54.2 |  |  |  |  |
| 12 | 37.1 | 3.16, dd (14.1, 5.5) | 1, 2, 13, 14/18 | 2 | 33 |
|  |  | 3.07, dd (14.0, 7.9) |  |  |  |
| 13 | 135.4 |  |  |  |  |
| 14/18 | 129.6 | 7.20, d (7.4) | 12, 16 | 15/17 | 2, 12a, 12b |
| 15/17 | 128.4 | 7.30, t (7.6) | 13 | 14/18, 16 |  |
| 16 | 126.4 | 7.24, m | 14/18 | 15/17 |  |
| 19 | 29.8^a^ | 2.04, m | 3, 4, 20, 21 | 4, 20, 21 |  |
| 20 | 16.7 | 0.69, d (6.8) | 4, 19, 21 | 19 | 4 |
| 21 | 17.8 | 0.83, d (6.8) | 4, 19, 20 | 19 | 4 |
| 22 | 170.3 |  |  |  |  |
| 23 | 114.3 |  |  |  |  |
| 24 | 150.5 |  |  |  |  |
| 25 | 126.9 |  |  |  |  |
| 26 | 125.1 | 8.26, d (7.9) | 24, 28 | 27 |  |
| 27 | 118.2 | 6.94, t (8.0) | 23, 25 | 26, 28 |  |
| 28 | 123.4 | 7.95, dd (8.2, 1.5) | 22, 24, 26 | 27 |  |
| 29 | 15.6 | 1.25, d (6.5) | 6, 7 | 7 | 6, 31 |
| 30 | 30.0^a^ | 2.04, m | 8, 9, 31, 32 | 9, 31, 32 |  |
| 31 | 17.6 | 0.89, d (6.8) | 9, 30, 32 | 30 | 29 |
| 32 | 17.6 | 0.89, d (6.8) | 9, 30, 31 | 30 |  |
| 33 | 21.2 | 1.35, s | 1, 10, 11, 34 |  |  |
| 34 | 21.2 | 1.22, s | 1, 10, 11, 33 |  |  |
| 6-NH |  | 9.20, d (8.0) | 22 | 6 |  |
| CHO | 160.4 | 8.34, d (1.9) | 25 | 25-NH |  |
| 24-OH |  | 12.72, br s |  |  |  |
| 25-NH |  | 9.82, d (2.1) | 24, 26, CHO | CHO |  |

^a^ values are exchangeable

## Table S6. NMR (600 MHz, CDCl_3_) data for 5

|  | Experimental δ_C_ (125 MHz) | Literature^a^ δ_C_ (100 MHz) | Experimental δ_H_ (600 MHz) | Literature^a^  δ_H_ (700 MHz) |
| --- | --- | --- | --- | --- |
| 1 | 79.2 | 79.0 | 3.19, br s | 3.23, s |
| 2 | 71.9 | 71.8 | 5.52, dd (9.6, 5.8) | 5.54, dd (6.0, 3.8) |
| 3 | 168.6 | 168.5 |  |  |
| 4 | 76.7 | 76.6 | 5.44, d (3.6) | 5.45, d (3.6) |
| 5 | 168.5 | 168.3 |  |  |
| 6 | 55.2 | 55.1 | 5.13, dd (8.8, 2.6) | 5.16, dd (8.8, 2.5) |
| 7 | 72.7 | 72.5 | 5.72, qd (6.4, 2.6) | 5.74, dd (6.5, 2.5) |
| 8 | 168.3 | 168.1 |  |  |
| 9 | 75.3 | 75.2 | 4.66, d (8.2) | 4.66, d (8.3) |
| 10 | 177.0 | 176.9 |  |  |
| 11 | 45.5 | 45.4 |  |  |
| 12 | 40.4 | 40.3 | 3.16, dd (14.0, 9.6) | 3.14, dd (14.0, 5.8) |
|  |  |  | 2.94, dd (14.0, 5.8) | 2.96, dd (14.0, 4.2) |
| 13 | 136.9 | 136.7 |  |  |
| 14/18 | 129.4 | 129.2 | 7.21, overlap | 7.22, t (8.0,8.5) |
| 15/17 | 128.8 | 128.6 | 7.27, overlap | 7.29, d (8.0) |
| 16 | 127.0 | 126.9 | 7.21, overlap | 7.22, m |
| 19 | 30.9 | 30.7 | 1.81, m | 1.83, m |
| 20 | 16.4 | 16.3 | 0.46, d (6.8) | 0.48, d (6.9) |
| 21 | 18.9 | 18.7 | 0.81, d (6.9) | 0.82, d (6.9) |
| 22 | 170.9 | 170.6 |  |  |
| 23 | 113.0 | 113.1 |  |  |
| 24 | 149.7 | 150.3 |  |  |
| 25 | n.o. | 129.2 |  |  |
| 26 | 114.7 | 115.8 | 6.96, d (8.0) | 7.05, d (7.5) |
| 27 | 119.0 | 118.9 | 6.75, t (7.8) | 6.78, t (7.5) |
| 28 | 118.7 | 119.6 | 6.86, dd (7.6) | 6.94, d (7.5) |
| 29 | 16.3 | 16.1 | 1.33, d (6.5) | 1.33, d (6.0) |
| 30 | 36.1 | 36.0 | 1.95, m | 1.96, m |
| 31 | 14.4 | 21.9 | 0.88, overlap | 1.33, d (6.0) |
| 32 | 24.9 | 24.7 | 1.51, m | 1.52, m |
|  |  |  | 1.20, m | 1.21, m |
| 33 | 27.0 | 26.8 | 1.40, s | 1.42, s |
| 34 | 22.0 | 21.9 | 1.30, s | 1.32, s |
| 35 | 10.7 | 10.5 | 0.88, overlap | 0.88, t (3.6) |
| 6-NH |  |  | 7.06, d (8.8) | not listed.^b^ |
| 24-OH |  |  | 12.05, br s | not listed.^b^ |

^a^ Li, X. *et al. Bioorg. Med. Chem. Lett.* 2013, *23*, 5123–5127

^n.o.^ Carbon resonances are not observed due to signal broadening

^b^ Value was not listed in the literature value but was observed in the spectrum acquired in our laboratory

## Table S7. NMR (600 MHz, CDCl_3_) data for 6

|  | Experimental δ_C_ (125 MHz) | Literature^a^ δ_C_ (100 MHz) | Experimental δ_H_ (600 MHz) | Literature^a^  δ_H_ (700 MHz) |
| --- | --- | --- | --- | --- |
| 1 | 79.2 | 79.0 | 3.19, d (12.0) | 3.20, s |
| 2 | 72.7 | 71.5 | 5.52, dd (9.5, 5.8) | 5.55, dd (6.0, 3.8) |
| 3 | 168.6 | 168.4 |  |  |
| 4 | 76.7 | 76.6 | 5.44, d (3.5) | 5.45, d (3.6) |
| 5 | 168.5 | 168.3 |  |  |
| 6 | 55.2 | 55.0 | 5.13, dd (8.9, 2.6) | 5.15, dd (8.5, 2.5) |
| 7 | 71.9 | 72.5 | 5.73, qd (6.5, 2.7) | 5.74, dd (6.6, 2.5) |
| 8 | 168.1 | 168.0 |  |  |
| 9 | 76.5 | 76.3 | 4.57, d (7.9) | 4.59, d (8.2) |
| 10 | 177.0 | 176.9 |  |  |
| 11 | 45.6 | 45.4 |  |  |
| 12 | 40.4 | 40.2 | 3.16, dd (14.0, 9.6) | 3.17, dd (14.0, 4.2) |
|  |  |  | 2.94, dd (14.0, 5.9) | 2.95, dd (14.0, 5.8) |
| 13 | 136.9 | 136.7 |  |  |
| 14/18 | 129.4 | 129.2 | 7.20, overlap | 7.21, t (8.0,8.5) |
| 15/17 | 128.8 | 128.6 | 7.27, overlap | 7.29, d (8.0) |
| 16 | 127.0 | 126.8 | 7.20, overlap | 7.22, m |
| 19 | 30.9 | 30.7 | 1.81, m | 1.84, m |
| 20 | 16.4 | 16.2 | 0.46, d (6.8) | 0.48, d (6.9) |
| 21 | 18.9 | 18.7 | 0.81, d (6.9) | 0.84, d (6.9) |
| 22 | 170.9 | 170.7 |  |  |
| 23 | n.o. | 112.9 |  |  |
| 24 | n.o. | 149.7 |  |  |
| 25 | n.o. | 129.1 |  |  |
| 26 | 114.7 | 114.7 | 6.96, dd (8.1, 1.4) | 6.98, d (7.5) |
| 27 | 119.0 | 118.8 | 6.75, t (7.9) | 6.78, t (7.5) |
| 28 | 118.6 | 118.6 | 6.86, dd (7.8, 1.3) | 6.89, d (7.5) |
| 29 | 16.3 | 16.1 | 1.33, d (6.6) | 1.33, d (6.3) |
| 30 | 30.2 | 30.0 | 2.12, dq (13.8, 6.8) | 2.15, m |
| 31 | 18.1 | 17.9 | 0.92, d (6.8) | 0.94, d (3.6) |
| 32 | 18.3 | 18.1 | 0.96, d (6.6) | 0.97, d (3.6) |
| 33 | 27.1 | 27.1 | 1.41, s | 1.44, s |
| 34 | 22.0 | 21.8 | 1.30, s | 1.34, s |
| 1-OH |  |  | 3.55, d (12.3) | not listed^b^ |
| 6-NH |  |  | 7.06, d (8.8) | not listed^b^ |
| 24-OH |  |  | 12.05, br s | not listed^b^ |

^a^ Li, X. *et al. Bioorg. Med. Chem. Lett.* 2013, *23*, 5123–5127

^b^ Value was not listed in the literature value but was observed in the spectrum acquired in our laboratory

^n.o.^ Carbon resonances are not observed due to signal broadening

## Table S8. NMR (600 MHz, CDCl_3_) data for 7

|  | Experimental *δ*_C_ (125 MHz) | Literature^a^ *δ*_C_ (100 MHz) | Experimental *δ*_H_ (600 MHz) | Literature^a^  *δ*_H_ (400 MHz) |
| --- | --- | --- | --- | --- |
| 1 | 79.2 | 79.0 | 3.20, d (10.4) | 3.20, d (12.4) |
| 2 | 71.9 | 71.8 | 5.52, dd (9.6, 5.7) | 5.52, dd (9.6, 5.9) |
| 3 | 168.4 | 168.3 |  |  |
| 4 | 76.8 | 76.7 | 5.44, d (3.5) | 5.45, d (3.5) |
| 5 | 168.4 | 168.3 |  |  |
| 6 | 55.3 | 55.2 | 5.13, dd (8.8, 2.6) | 5.14, dd (6.5, 2.6) |
| 7 | 72.5 | 72.3 | 5.74, qd (6.4, 2.6) | 5.74, dd (6.5, 2.6) |
| 8 | 168.2 | 168.1 |  |  |
| 9 | 75.2 | 75.1 | 4.66, br d (8.3) | 4.66, d (8.3) |
| 10 | 177.0 | 176.9 |  |  |
| 11 | 45.5 | 45.4 |  |  |
| 12 | 40.4 | 40.2 | 3.16, dd (14.0, 9.5) | 3.16, dd (13.9, 9.6) |
|  |  |  | 2.94, dd (14.1, 5.8) | 2.93, dd (13.9, 5.6) |
| 13 | 136.9 | 136.8 |  |  |
| 14/18 | 129.3 | 129.2 | 7.26, overlap | 7.19-7.29 |
| 15/17 | 128.7 | 128.6 | 7.21, overlap | 7.19-7.29 |
| 16 | 127.0 | 126.8 | 7.21, overlap | 7.19-7.29 |
| 19 | 30.8 | 30.7 | 1.81, m | 1.80, m |
| 20 | 16.2 | 16.1 | 0.46, d (6.9) | 0.45, d (6.9) |
| 21 | 18.8 | 18.7 | 0.81, d (6.9) | 0.81, d (6.9) |
| 22 | 170.3 | 170.2 |  |  |
| 23 | 113.0 | 113.0 |  |  |
| 24 | 150.7 | 150.6 |  |  |
| 25 | 127.5 | 127.3 |  |  |
| 26 | 124.9 | 124.8 | 8.54, dd (8.2, 2.7) | 8.54, dd (8.0, 1.2) |
| 27 | 119.1 | 118.9 | 6.93, t (8.2) | 6.93, t (8.0) |
| 28 | 120.5 | 120.5 | 7.34, dd (8.2, 1.3) | 7.34, dd (8.0, 1.2) |
| 29 | 16.4 | 16.3 | 1.34, d (6.6) | 1.34, d (6.5) |
| 30 | 36.1 | 36.0 | 1.97, m | 1.96, m |
| 31 | 14.4 | 14.2 | 0.88, overlap | 0.89, d (6.8) |
| 32 | 24.9 | 24.7 | 1.51, m | 1.52, m |
|  |  |  | 1.20, m | 1.21, m |
| 33 | 27.0 | 26.9 | 1.41, s | 1.41, s |
| 34 | 22.0 | 21.9 | 1.31, s | 1.31, s |
| 35 | 10.7 | 10.5 | 0.88, overlap | 0.88, t (7.4) |
| CHO | 159.2 | 159.3 | 8.50, br s | 8.50, d (1.7) |
| 1-OH |  |  | 3.56, d (12.4) | 3.57, d (12.4) |
| 6-NH |  |  | 7.21, overlap | 7.19-7.29 |
| 24-OH |  |  | 12.63, br s | 12.63, br s |
| 25-NH |  |  | 7.99, br s | 8.01, s |

^a^ Takeda, Y. *et al. J. Nat. Prod.* 1998, *61*, 978-981

## Table S9. NMR (600 MHz, CDCl_3_) data for 8

|  | Experimental δ_C_ (125 MHz) | Literature^a^ δ_C_ (175 MHz) | Experimental δ_H_ (600 MHz) | Literature^a^  δ_H_ (700 MHz) |
| --- | --- | --- | --- | --- |
| 1 | 79.2 | 79.2 | 3.20, d (12.2) | 3.16, s |
| 2 | 72.0 | 71.9 | 5.53, dd (9.9, 5.8) | 5.53, dd (6.0, 4.0) |
| 3 | 168.4 | 168.8 |  |  |
| 4 | 76.9 | 76.7 | 5.44, d (3.6) | 5.44, d (3.6) |
| 5 | 168.4 | 168.7 |  |  |
| 6 | 55.4 | 55.2 | 5.13, dd (8.8, 2.6) | 5.12, dd (8.5, 2.5) |
| 7 | 72.5 | 72.7 | 5.74, qd (6.4, 2.6) | 5.73, dd (6.5, 2.5) |
| 8 | 168.1 | 168.0 |  |  |
| 9 | 76.5 | 76.5 | 4.58, d (7.8) | 4.58, dd (8.0) |
| 10 | 177.1 | 175.9 |  |  |
| 11 | 45.6 | 45.6 |  |  |
| 12 | 40.4 | 40.4 | 3.16, dd (14.0, 9.6) | 3.16, dd (14.0, 4.2) |
|  |  |  | 2.94, dd (14.0, 5.8) | 2.98, dd (14.0, 5.8) |
| 13 | 136.9 | 137.3 |  |  |
| 14/18 | 129.4 | 129.0 | 7.21, m | 7.20, t (8.5, 8.0) |
| 15/17 | 128.8 | 128.2 | 7.27, overlap | 7.29, d (8.0) |
| 16 | 127.0 | 126.3 | 7.21, m | 7.23, m |
| 19 | 30.9 | 30.9 | 1.81, m | 1.80, m |
| 20 | 16.5 | 18.9 | 0.46, d (6.8) | 0.46, d (6.9) |
| 21 | 18.8 | 16.4 | 0.81, d (6.9) | 0.81, d (6.9) |
| 22 | 170.4 | 170.4 |  |  |
| 23 | 113.0 | 114.6 |  |  |
| 24 | 150.7 | 150.6 |  |  |
| 25 | 127.5 | 126.7 |  |  |
| 26 | 124.9 | 125.1 | 8.56, dd (8.0, 1.4) | 8.58, d (7.5) |
| 27 | 119.1 | 118.1 | 6.94, t (8.1) | 6.94, t (7.5) |
| 28 | 120.4 | 122.9 | 7.33, dd (8.3, 1.4) | 7.37, d (7.5) |
| 29 | 16.3 | 16.3 | 1.34, d (6.5) | 1.33, d (6.0) |
| 30 | 30.2 | 30.2 | 2.13, m | 2.17, m |
| 31 | 18.3 | 18.3 | 0.97, d (6.7) | 0.97, d (3.6) |
| 32 | 18.1 | 18.1 | 0.92, d (6.8) | 0.92, d (3.6) |
| 33 | 27.1 | 27.4 | 1.42, s | 1.41, s |
| 34 | 22.0 | 22.0 | 1.31, s | 1.30, s |
| CHO | 159.1 | 160.8 | 8.50, br d (1.7) | 8.51, br s |
| 1-OH |  |  | 3.55, d (12.3) | not listed^b^ |
| 6-NH |  |  | 7.16, d (8.8) | not listed^b^ |
| 24-OH |  |  | 12.63, br s | not listed^b^ |
| 25-NH |  |  | 7.91, br s | 7.92, br s |

^a^ X. Li *et al. Bioorg. Med. Chem. Lett.* 2013, *23*, 5123-5127

^b^ Value was not listed in the literature value but was observed in the spectrum acquired in our laboratory

## Table 10. ^1^H NMR (600 MHz CDCl_3_) Data for the (*S*)- and (*R*)-MTPA Esters of 9

|  | *S*-MTPA ester *δ*_H_ (multiplicity, *J* = Hz) | *R*-MTPA ester *δ*_H_ (multiplicity, *J* = Hz) | *Δδ (δ_S_ - δ_R_)* values |
| --- | --- | --- | --- |
| 2-Me | 1.34, s | 1.33, s | +0.01 |
|  | 1.12, s | 1.06, s | +0.06 |
| 3 | 5.47, d (3.8) | 5.51, d (3.7) | -0.04 |
| 4 | 4.83, dt (9.4, 3.8) | 4.89, dt (9.4, 3.8) | -0.06 |
| 5 | 2.78, dd (14.7, 9.4) | 2.82, dd (14.7, 9.4) | -0.04 |
|  | 2.69, dd (14.9, 4.0) | 2.76, dd (14.7, 3.8) | -0.07 |
| 7/11 | 7.13, d (6.8) | 7.18, d (7.4) | -0.05 |
| 8/10 | 7.48, m | 7.48, m |  |
| 9 | 7.53, m | 7.53, m |  |

## Figure S1a ^1^H NMR spectrum of 1 in DMSO-*d*_6_

## Figure S1b ^13^C NMR spectrum of 1 in DMSO-*d*_6_

## Figure S1c HSQC spectrum of 1 in DMSO-*d*_6_

__

## Figure S1d HMBC spectrum of 1 in DMSO-*d*_6_

## Figure S1e ^1^H-^1^H COSY spectrum of 1 in DMSO-*d*_6_

## Figure S1f ROESY spectrum of 1 in DMSO-*d*_6_

## Figure S2a ^1^H NMR spectrum of 2 in DMSO-*d*_6_

## Figure S2b ^13^C NMR spectrum of 2 in DMSO-*d*_6_

## Figure S2c HSQC spectrum of 2 in DMSO-*d*_6_

__

## Figure S2d HMBC spectrum of 2 in DMSO-*d*_6_

__

## Figure S2e ^1^H-^1^H COSY spectrum of 2 in DMSO-*d*_6_

## Figure S2f ROESY spectrum of 2 in DMSO-*d*_6_

## Figure S3a ^1^H NMR spectrum of 3 in DMSO-*d*_6_

## Figure S3b ^13^C NMR spectrum of 3 in DMSO-*d*_6_

## Figure S4a ^1^H NMR spectrum of 4 in DMSO-*d*_6_

## Figure S4b ^13^C NMR spectrum of 4 in DMSO-*d*_6_

## Figure S5a ^1^H NMR spectrum of 5 in CDCl_3_

## Figure S5b ^13^C NMR spectrum of 5 in CDCl_3_

__

## Figure S5c HETLOC spectrum of 5 in CDCl_3_

## Figure S6a ^1^H NMR spectrum of 6 in CDCl_3_

## Figure S6b ^13^C NMR spectrum of 6 in CDCl_3_


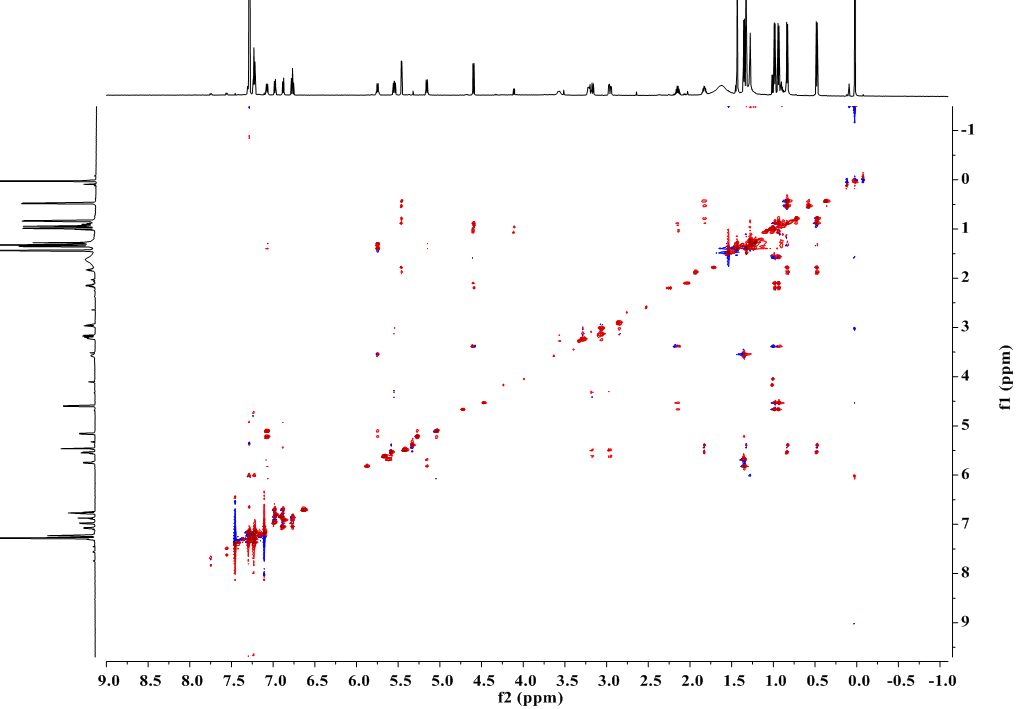


## Figure S6c HETLOC spectrum of 6 in CDCl_3_

## Figure S7a ^1^H NMR spectrum of 7 in CDCl_3_

_
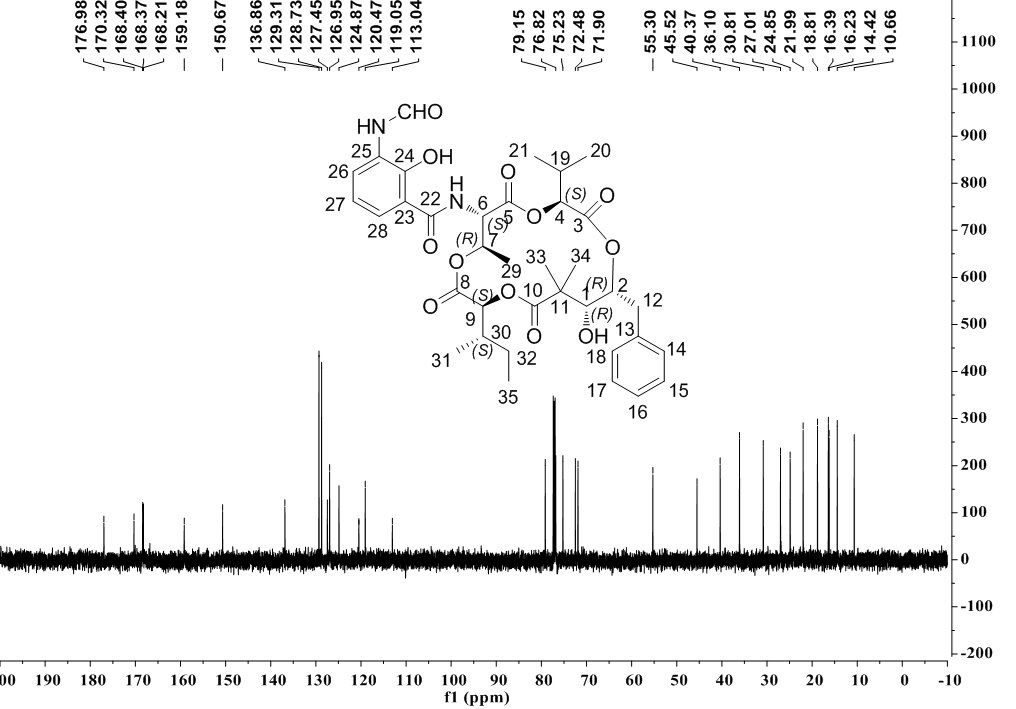
_

## Figure S7b ^13^C NMR spectrum of 7 in CDCl_3_

_
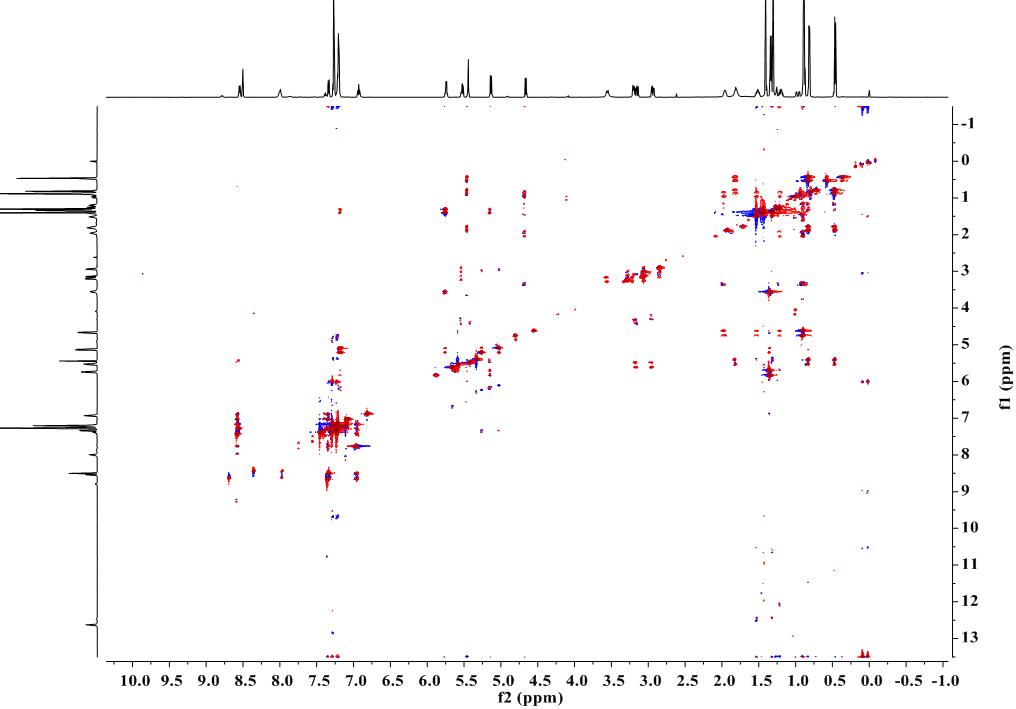
_

## Figure S7c HETLOC spectrum of 7 in CDCl_3_

## Figure S8a ^1^H NMR spectrum of 8 in CDCl_3_

## Figure S8b ^13^C NMR spectrum of 8 in CDCl_3_

## Figure S8c HETLOC spectrum of 8 in CDCl_3_

## Figure S9a ^1^H NMR spectrum of 9 in CDCl_3_

## Figure S9b ^1^H NMR spectrum of *S*-MTPA ester of 9 in CDCl_3_

_
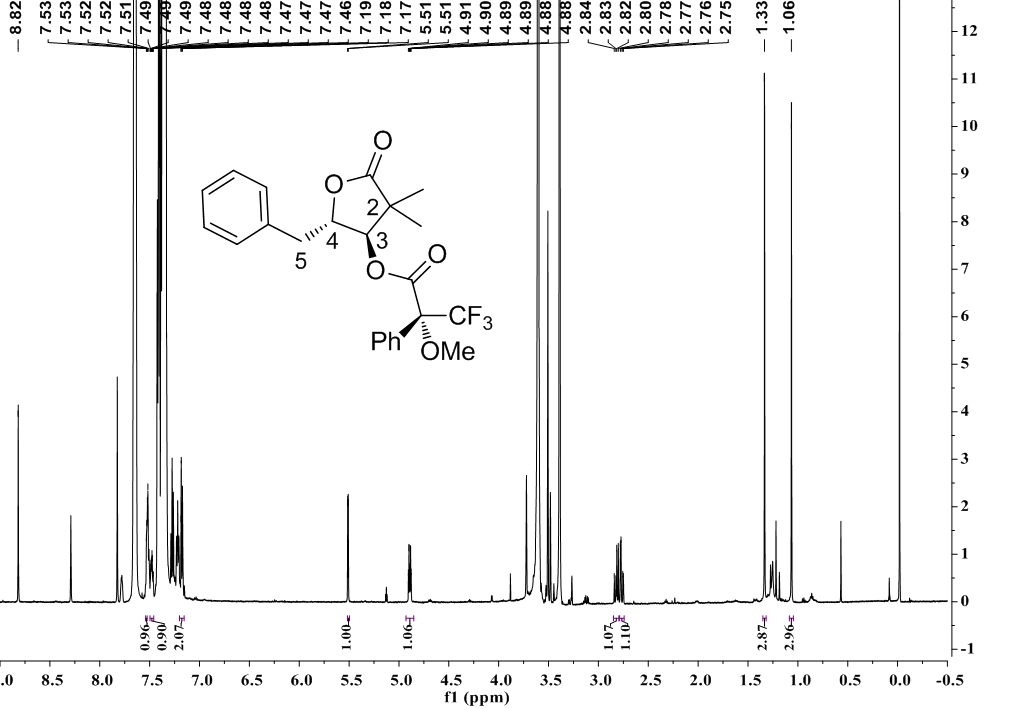
_

## Figure S9c ^1^H NMR spectrum of *R*-MTPA ester of 9 in CDCl_3_

_
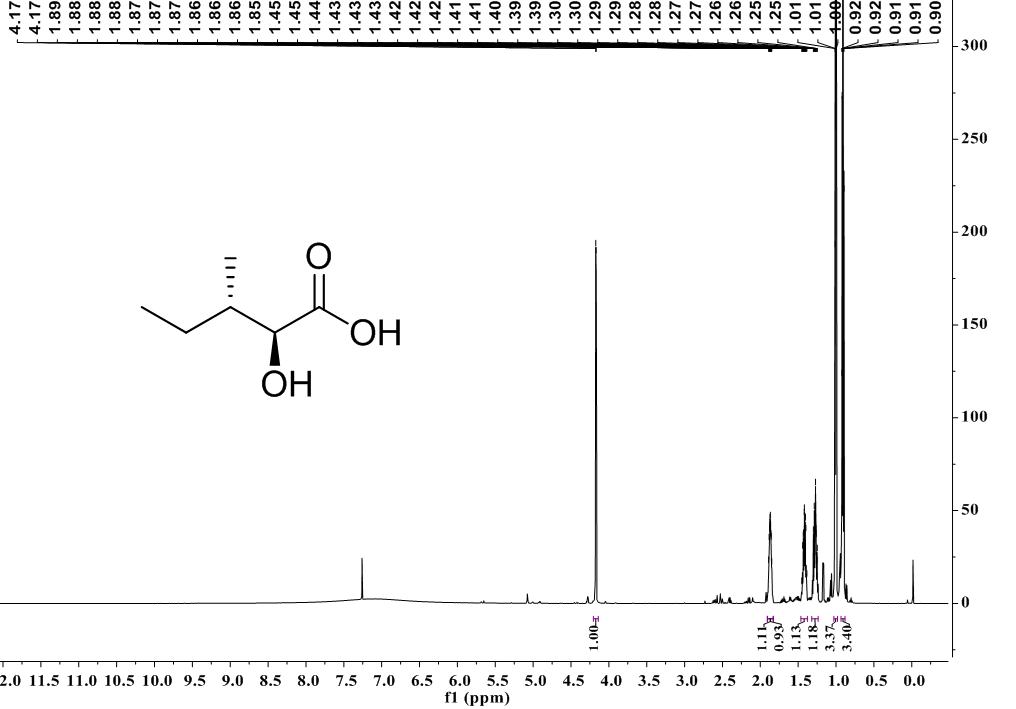
_

## Figure S10a ^1^H NMR spectrum of 10 in CDCl_3_

_
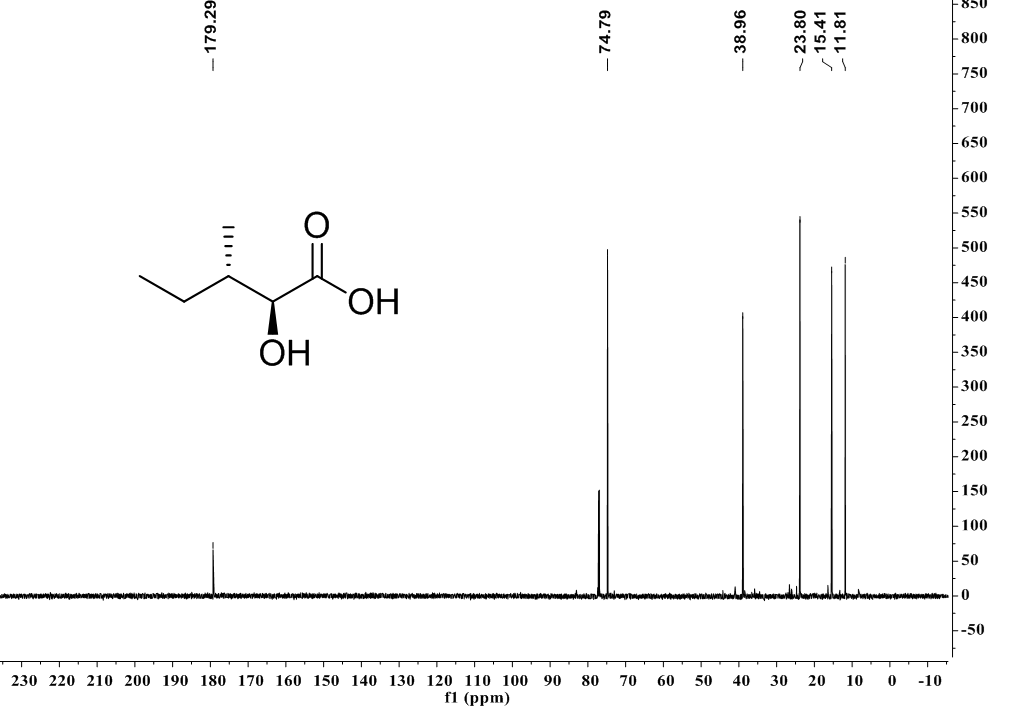
_

## Figure S10b ^13^C NMR spectrum of 10 in CDCl_3_


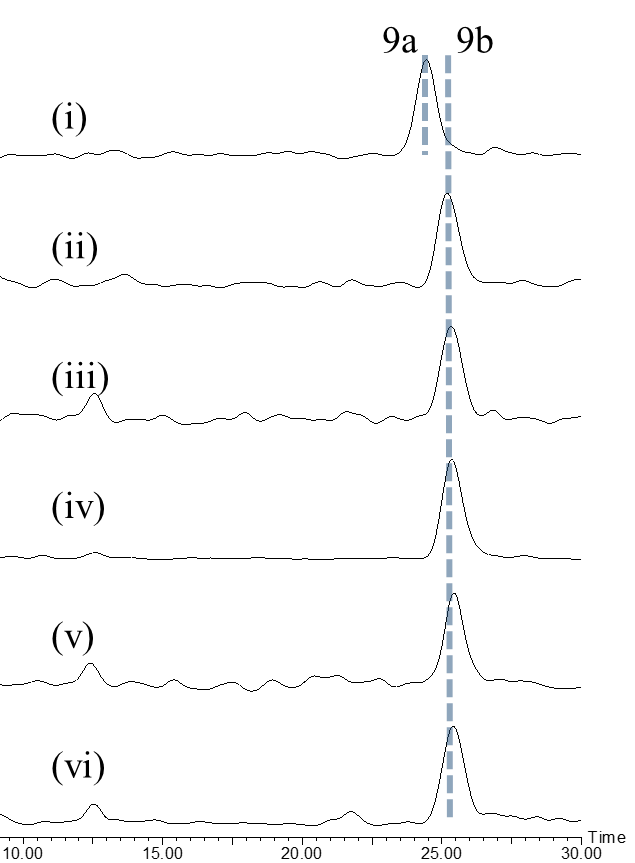


## Figure S11a. LCMS chromatogram of extracted ion at 437 [M + H]^+^from Mosher’s esterification reaction

(i) **9** reacted with *S*-MTPA-Cl to give **9a**, (ii) **9** reacted with *R*-MTPA-Cl to give **9b**, (iii) Hydrolysate of **7** reacted with *R*-MTPA-Cl, (iv) Hydrolysate of **8** reacted with *R*-MTPA-Cl, (v) Hydrolysate of **5** reacted with *R*-MTPA-Cl, (vi) Hydrolysate of **6** reacted with *R*-MTPA-Cl


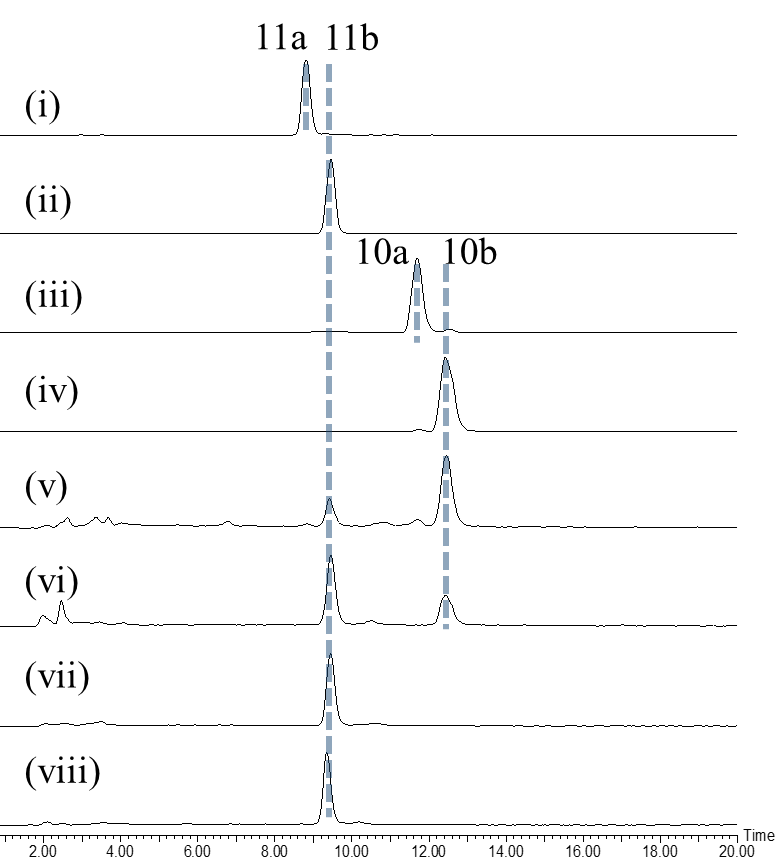


## Figure S11b. LC-MS chromatogram of extracted ion at 333 [M - H] and 347 [M - H]^-^ from Mosher’s esterification reaction

(i) **11** reacted with *S*-MTPA-Cl to give **11a**, (ii) **11** reacted with *R*-MTPA-Cl to give **11b**, (iii) **10** reacted with *S*-MTPA-Cl to give **10a**, (iv) **10** reacted with *R*-MTPA-Cl to give **10b**, (v) Hydrolysate of **7** reacted with *R*-MTPA-Cl, (vi) Hydrolysate of **5** reacted with *R*-MTPA-Cl, (vii) Hydrolysate of **6** reacted with *R*-MTPA-Cl, (viii) Hydrolysate of **8** reacted with *R*-MTPA-Cl

**
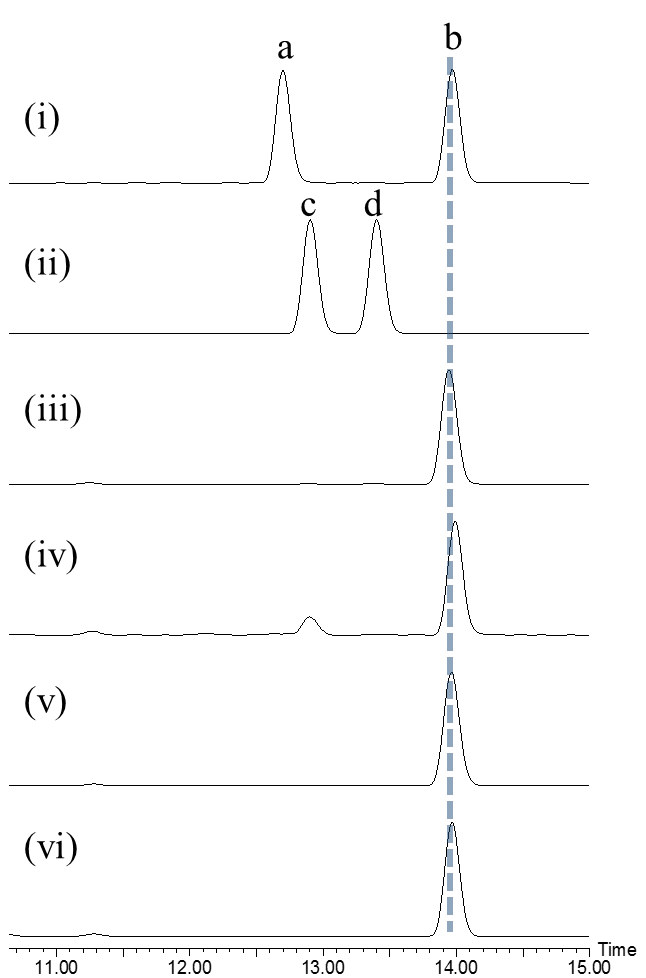
**

## Figure S12. LC-MS chromatogram of extracted ion at 414 [M + H]^+^ from FDLA derivatization reaction

(i) Standard *L*-threonine reacted with *L*-FDLA or *D*-FDLA to give (a) *L*-FDAA-*L*-Thr, (b) *D*-FDAA- *L*-Thr, (ii) Standard *L*-*allo*-Thr reacted with *L*-FDLA or *D*-FDLA to give (a) *L*-FDAA-*L*-*allo*-Thr, (b) *D*-FDAA- *L*-*allo*-Thr, (iii) Hydrolysate of **7** reacted with *D*-FDLA, (iv) Hydrolysate of **8** reacted with *D*-FDLA, (v) Hydrolysate of **5** reacted with *D*-FDLA, (vi) Hydrolysate of **6** reacted with *D*-FDLA


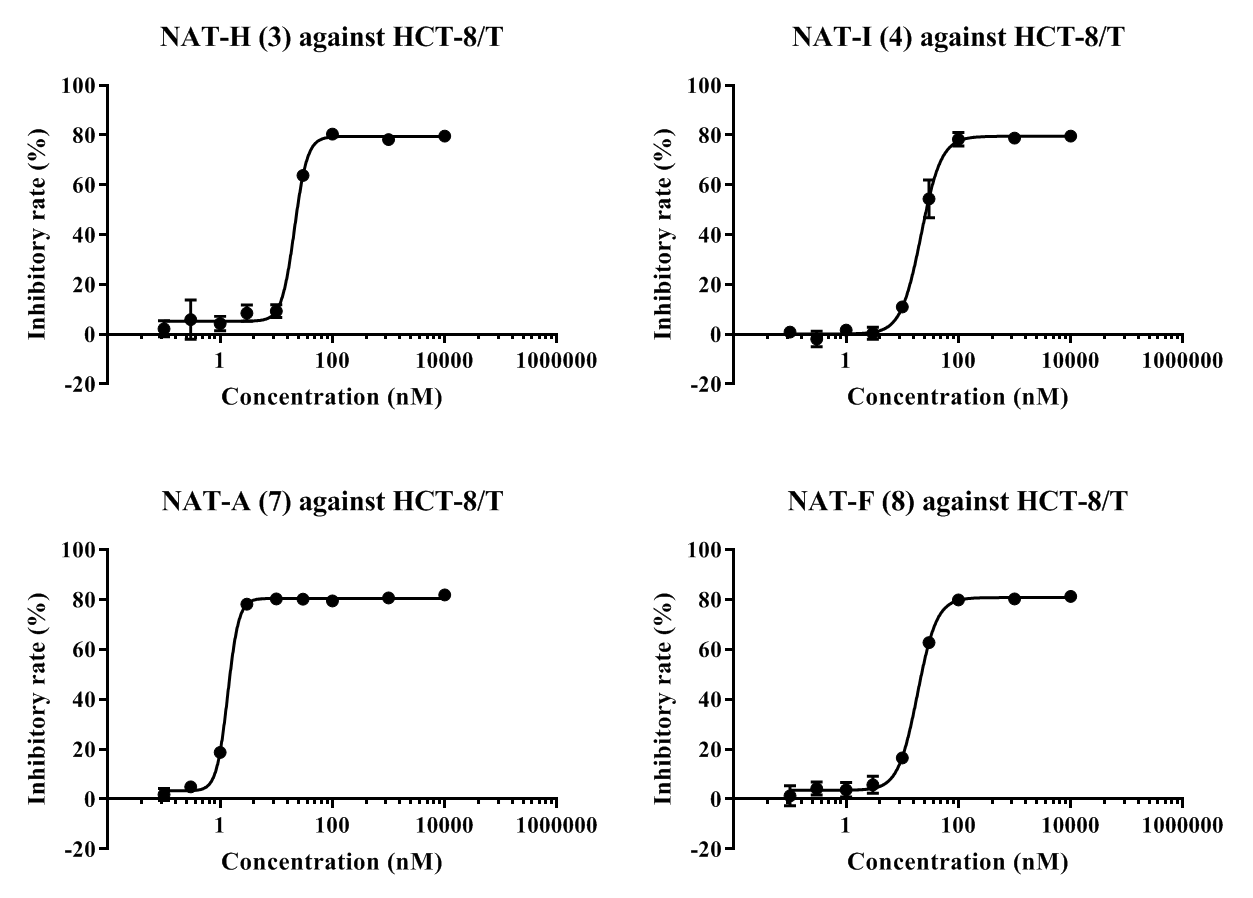


## Figure S13. Graphs for CCK-8 cytotoxicity assays in HCT-8/T cell line


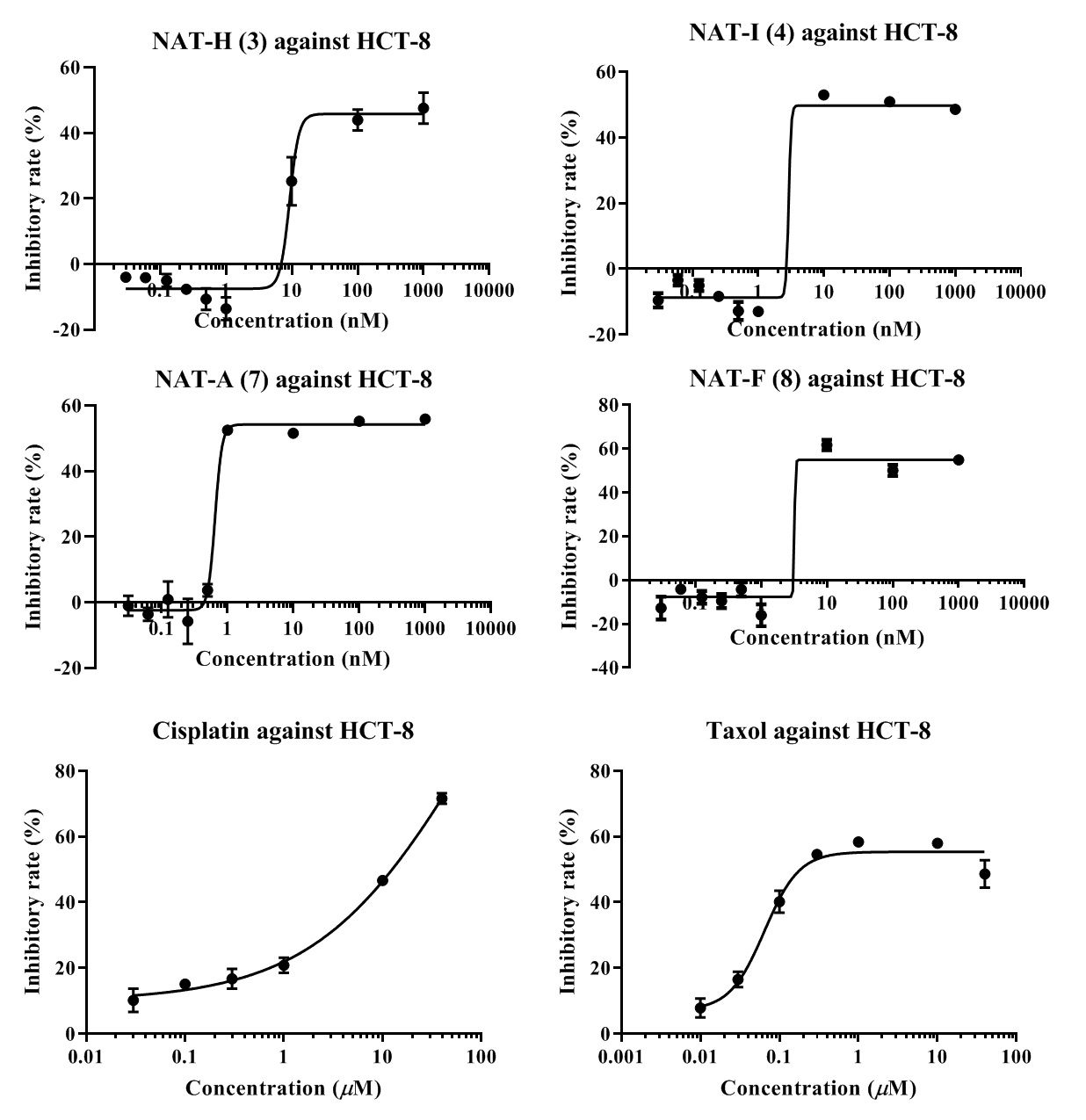


## Figure S14. Graphs for CCK-8 cytotoxicity assays in HCT-8 cell line


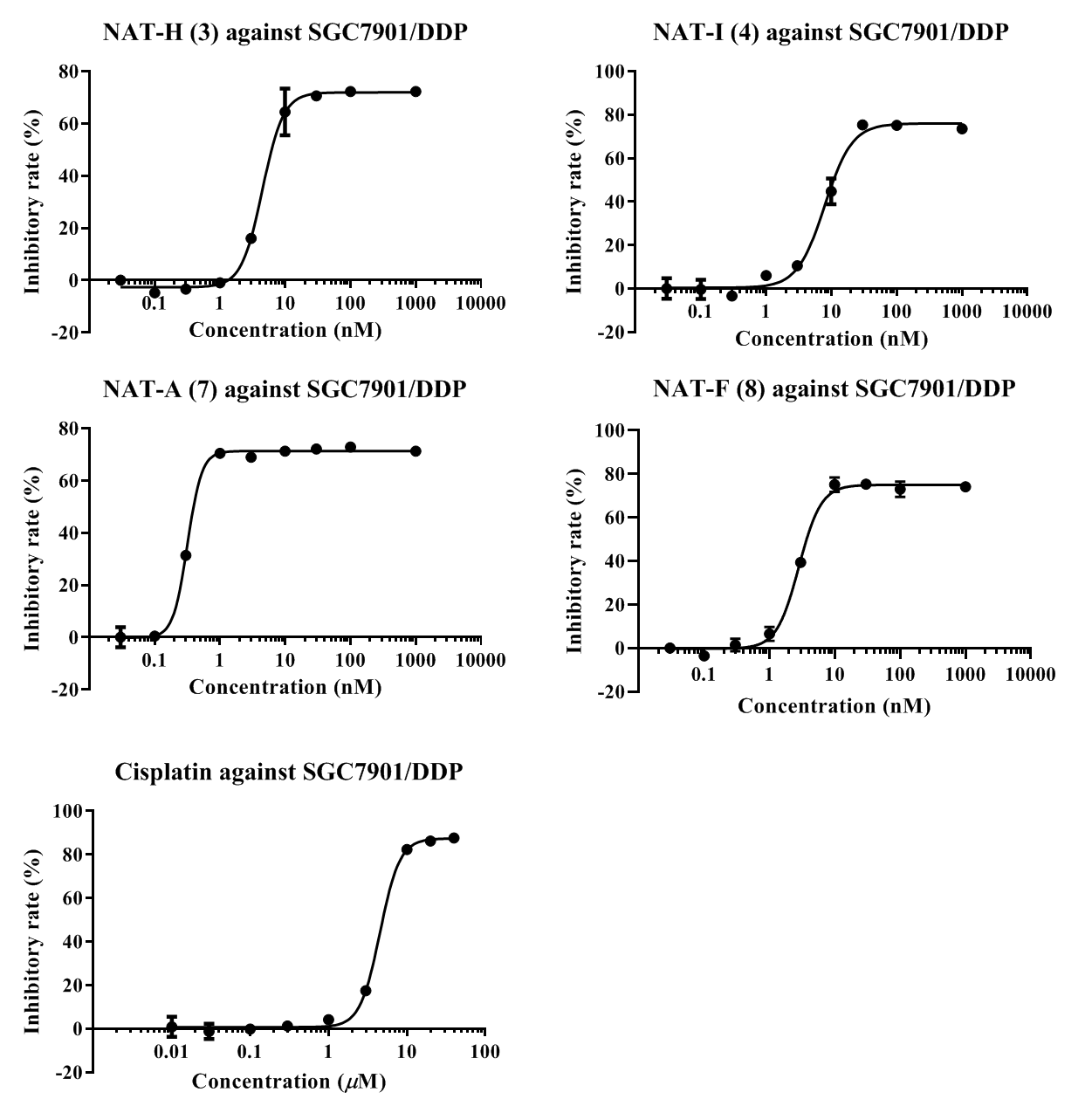


## Figure S15. Graphs for CCK-8 cytotoxicity assays in SGC7901/DDP cell line


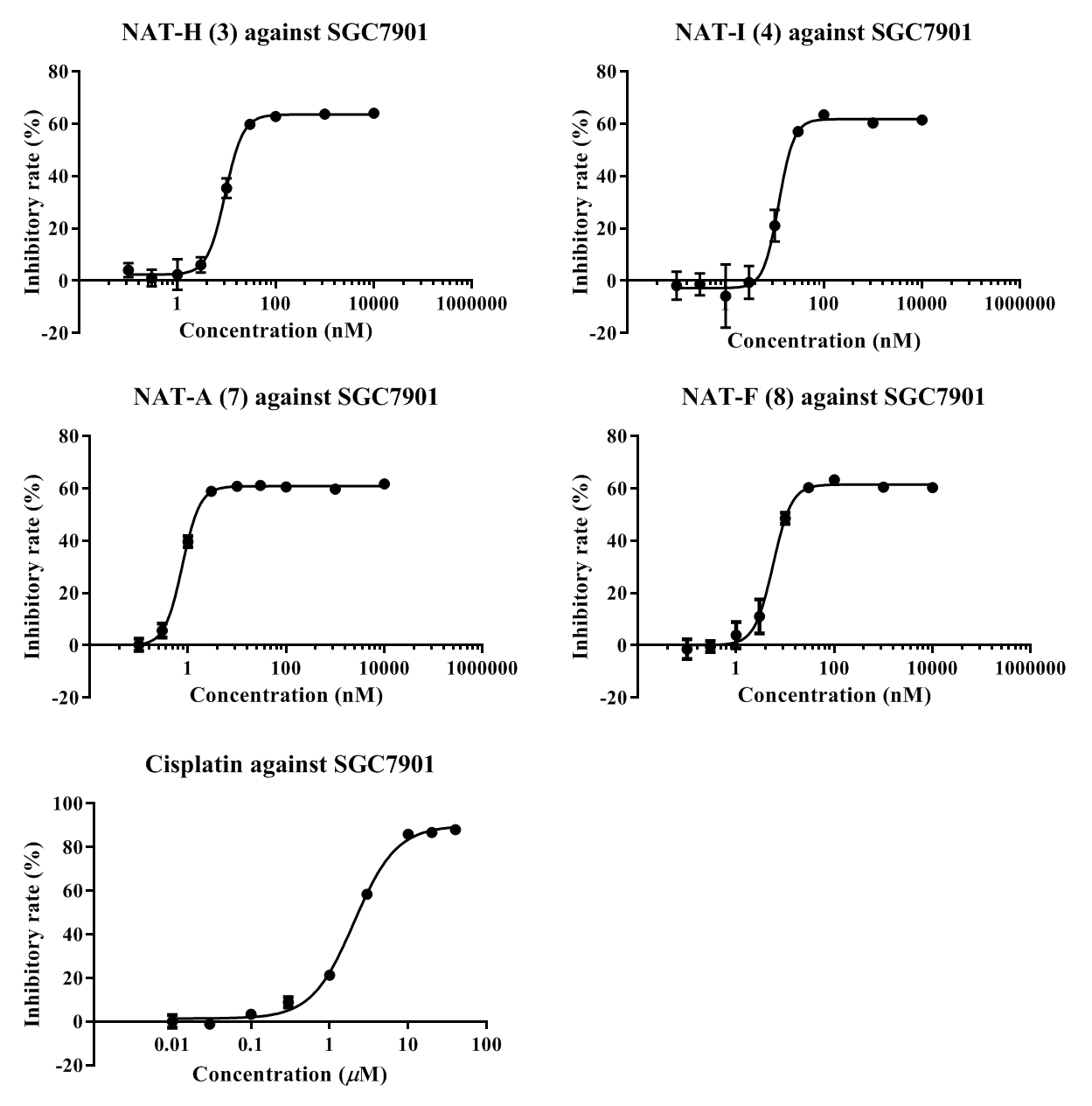


## Figure S16. Graphs for CCK-8 cytotoxicity assays in SGC7901 cell line


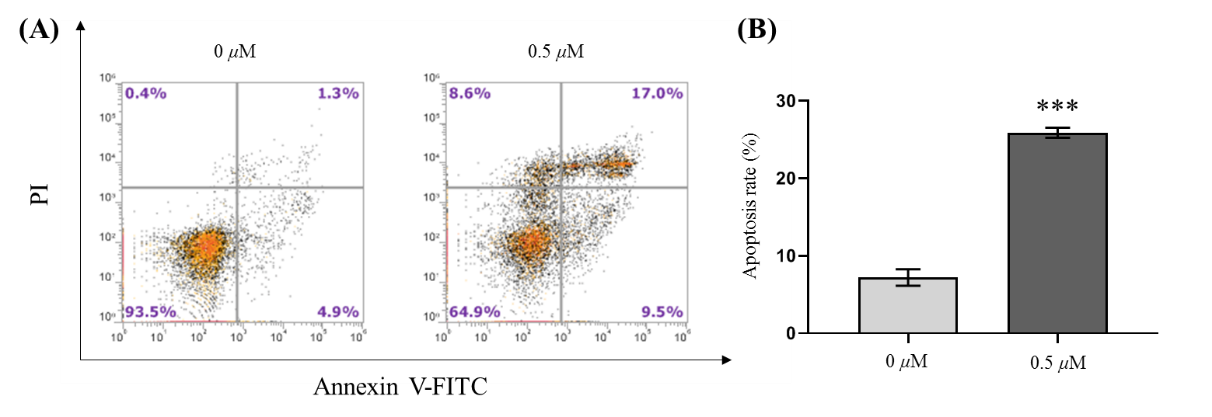


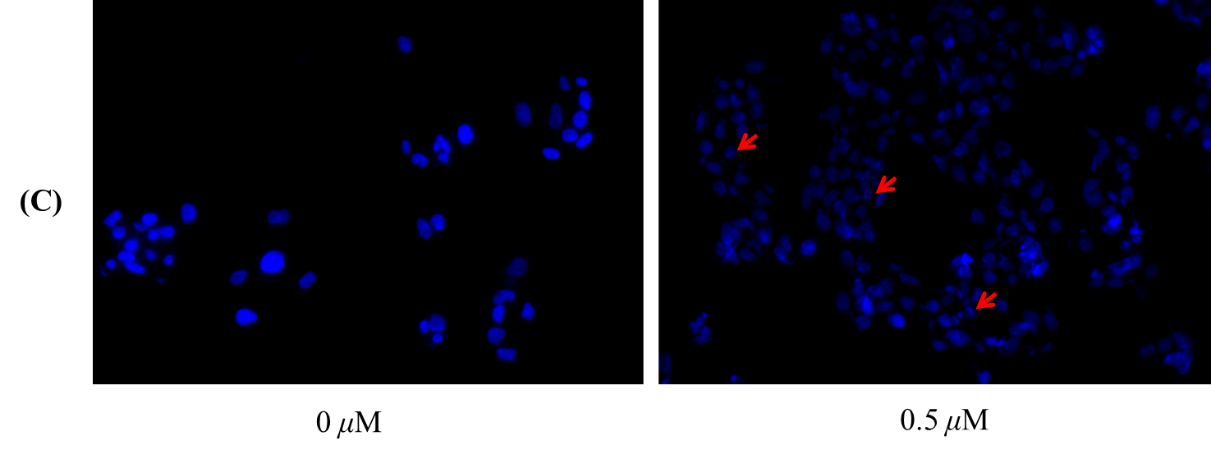


## Figure S17. Effects of NAT-A (3) on cell proliferation and apoptosis in HCT-8 cells.

(A) After administration with NAT-A (0 and 0.5 *μ*M) for 72 h, HCT-8 cells were stained with Annexin V/PI and the apoptosis level was analyzed by flow cytometry. (B) Quantitative analysis of Annexin V/PI double-staining. The percentage of Annexin V-positive cells was represented in bar charts as mean ± SD of three replicates (***P < 0.001) (C) Nucleolus morphologic changes observed by fluorescent microscope (200×). Apoptotic cells were observed for apoptotic bodies and nuclei pyknosis.
